# Sensitivity of CD3/CD28-stimulated *versus* non-stimulated lymphocytes to ionizing radiation and genotoxic anticancer drugs: key role of ATM in the differential radiation response

**DOI:** 10.1038/s41419-018-1095-7

**Published:** 2018-10-15

**Authors:** Daniel Heylmann, Jennifer Badura, Huong Becker, Jörg Fahrer, Bernd Kaina

**Affiliations:** 1 Institute of Toxicology, University Medical Center, Obere Zahlbacher Strasse 67, 55131 Mainz, Germany; 2 Present Address: Rudolf Buchheim Institute of Pharmacology, Justus Liebig University Giessen, Schubertstraße 81, 35392 Giessen, Germany

## Abstract

Activation of T cells, a major fraction of peripheral blood lymphocytes (PBLCS), is essential for the immune response. Genotoxic stress resulting from ionizing radiation (IR) and chemical agents, including anticancer drugs, has serious impact on T cells and, therefore, on the immune status. Here we compared the sensitivity of non-stimulated (non-proliferating) vs. CD3/CD28-stimulated (proliferating) PBLC to IR. PBLCs were highly sensitive to IR and, surprisingly, stimulation to proliferation resulted in resistance to IR. Radioprotection following CD3/CD28 activation was observed in different T-cell subsets, whereas stimulated CD34+ progenitor cells did not become resistant to IR. Following stimulation, PBLCs showed no significant differences in the repair of IR-induced DNA damage compared with unstimulated cells. Interestingly, ATM is expressed at high level in resting PBLCs and CD3/CD28 stimulation leads to transcriptional downregulation and reduced ATM phosphorylation following IR, indicating ATM to be key regulator of the high radiosensitivity of resting PBLCs. In line with this, pharmacological inhibition of ATM caused radioresistance of unstimulated, but not stimulated, PBLCs. Radioprotection was also achieved by inhibition of MRE11 and CHK1/CHK2, supporting the notion that downregulation of the MRN-ATM-CHK pathway following CD3/CD28 activation results in radioprotection of proliferating PBLCs. Interestingly, the crosslinking anticancer drug mafosfamide induced, like IR, more death in unstimulated than in stimulated PBLCs. In contrast, the bacterial toxin CDT, damaging DNA through inherent DNase activity, and the DNA methylating anticancer drug temozolomide induced more death in CD3/CD28-stimulated than in unstimulated PBLCs. Thus, the sensitivity of stimulated vs. non-stimulated lymphocytes to genotoxins strongly depends on the kind of DNA damage induced. This is the first study in which the killing response of non-proliferating vs. proliferating T cells was comparatively determined. The data provide insights on how immunotherapeutic strategies resting on T-cell activation can be impacted by differential cytotoxic effects resulting from radiation and chemotherapy.

## Introduction

The adaptive immune response is based on a complex scenario of lymphocyte activation^1^ involving T cells, which represent the major fraction in peripheral blood lymphocytes (PBLCs) (70–90 %)^2^. Once stimulated through the CD3 receptor and co-receptors by antigens on the surface of antigen-presenting cells, T cells start to reprogram gene expression, proliferate, and elicit a pathogen-specific immune response. This occurs in the lymph nodes, thymus, spleen, and during inflammatory processes in target tissues^3^. Notably, the tumor environment is heavily infiltrated by T cells, which can be stimulated by tumor antigens^4^. Immune cell infiltration in the tumor has a high prognostic importance as to tumor progression and patient’s survival in many cancer diseases^5^.

In cancer radiotherapy, tumor-infiltrated lymphocytes are strongly affected by ionizing radiation (IR)^6^. IR (e.g., X-rays and γ-rays) directly ionizes atoms and molecules in the DNA resulting in bio-radicals^7^. This leads to fragmentations of C–C and C–O bonds that give rise to DNA single-strand breaks (SSBs) and double-strand breaks (DSBs), which are main toxic lesions^8,9^. IR also generates highly reactive radicals that damage indirectly DNA and other biomolecules^10,11^. Human beings are exposed to IR daily from natural terrestrial and cosmic irradiation, and also, with higher risk, if they live near nuclear waste territories, e.g., uranium mining districts^12,13^. Residents and clean-up workers are also in close contact to IR after nuclear disasters as Chernobyl or Fukushima^14,15^. In particular, the hematopoietic system is strongly affected by IR. Besides hematopoietic stem cells, especially T cells such cytotoxic T cells (CTLs) and T-helper cells (Th) were reported to be highly radiosensitive^16^. It is well known that radiotherapy leads to immunosuppressive side effects and leucopenia in patients, which is also apparent in the so-called acute radiation sickness^17–20^.

In cancer therapy, IR is frequently combined with chemotherapy^21,22^, in order to enhance the therapeutic effect. This is also achieved by combining immunotherapy settings such as adoptive T-cell transfer or dendritic cell (DC) vaccination in combination with radiotherapy, chemotherapy, and small inhibitory molecules, e.g., the poly(ADP) ribosyltransferase 1 (PARP) inhibitor olaparib^23–32^. Genotoxicants used in classical chemotherapy are, e.g., the methylating agent temozolomide (TMZ), which is used in combination with radiation in glioblastoma therapy, and the DNA crosslinking drug cyclophosphamide, which is widely used as anticancer drug and, at lower doses, as immunosuppressing agent^33,34^. There are several radiomimetic drugs, including the bacterial toxin cytolethal distending toxin (CDT), which cleaves DNA yielding DNA DSBs through a DNase I-like subunit^35^.

In cancer immunotherapy, stimulation of CTLs through tumor peptides plays a major role. It can also be conducted by transfer of antigen-specific T cells. Stimulated immune cells start an antitumoral response and secrete perforins, granzymes, and Fas ligands^36–38^. As radio- and chemotherapy target not only tumor cells, but also T cells, which have the potential to infiltrate in the tumor environment and thus have an impact on the tumor response^4,39,40^, it is of importance to compare the radiosensitivity of unstimulated lymphocytes, circulating in the periphery, and stimulated lymphocytes, being active at sites of inflammation and the tumor microenvironment. Here we present a comparative study on the radiation response of unstimulated, non-proliferating and CD3/CD28-stimulated, proliferating PBLCs, and T-cell subpopulations obtained from human healthy volunteers. Furthermore, we studied DNA repair and DNA damage-triggered cell signaling, and compared the effect of IR with TMZ, the activated form of cyclophosphamide, and CDT on cell death induction.

## Results

**Fig. 1:**
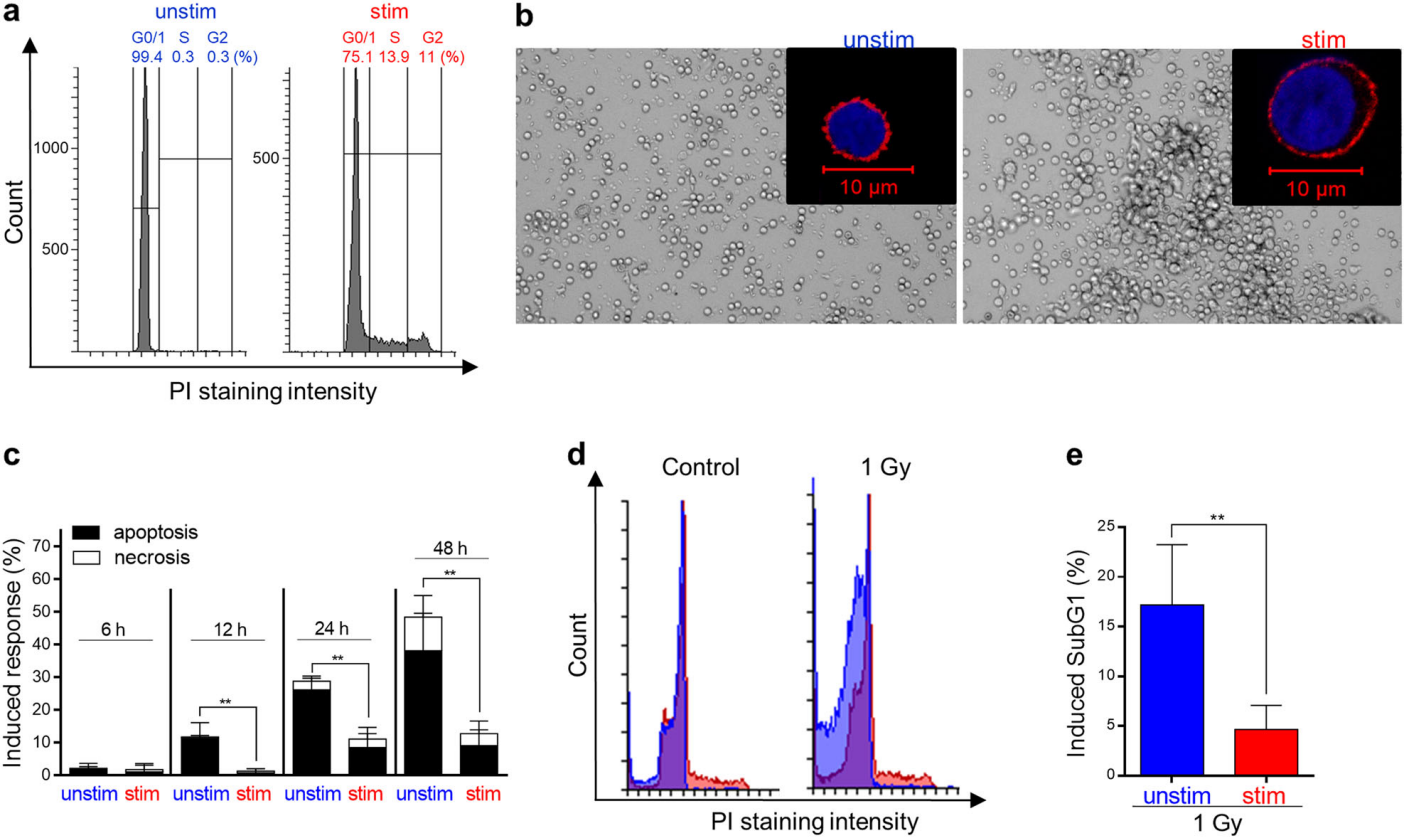
Radiation response of unstimulated vs. CD3-/CD28-stimulated PBLCs. **a** Cell cycle distribution (PI staining, flow cytometry) showed unstimulated cells only in G0/1. Stimulation for 48 h induced proliferation of PBLC as shown by an increase of cells in S and G2 phase (and by increased CD25 expression as shown in Figure S3c). **b** Stimulated PBLCs showed an increase in cell size and changes in morphology as determined by light microscopy and staining of nuclei (blue), and CD3 T-cell receptor (red) by immunohistochemistry (LSM). An increase in cell size was also visible in forward and sideward scatter by flow cytometry as shown in Figure S3d. **c** Unstimulated PBLCs underwent significantly more cell death (mainly apoptosis) 12–48 h after 1 Gy compared with stimulated PBLs (annexinV/PI staining, *n* = 3–4, mean values, SD, *t*-test concerning apoptotic part **p < 0.01). Stimulation-induced radioprotection was also obvious 72 h after 0.5 and 1 Gy (Figure S3e). **d**, **e** Stimulation of PBLs also resulted 24 h after 1 Gy in less DNA fragmentation as revealed by SubG1 flow cytometry (*n* = 4, mean value, SD, *t*-test **p < 0.01)

Throughout this study, we compared unstimulated vs. stimulated PBLCs from the same donor (paired samples). Unstimulated PBLCs, containing following purification mainly T cells, are arrested in G0/G1. Upon stimulation of the CD3 T-cell receptor and the CD28 co-receptor with anti-CD3 and anti-CD28, respectively, the cells start to proliferate, as shown by the emergence of S and G2 cells in the population (Fig. 1a; see also Supplement Fig. S3c). Upon stimulation, T cells (identified by CD3 staining) underwent morphological changes and increased in size (Fig. 1b and Supplement Fig. S3d).

To determine cell death induced by radiation of unstimulated vs. stimulated PBLCs, we irradiated the cells and measured the death fraction by annexinV/propidium iodide (PI). The experiments revealed that the majority of cells underwent apoptosis, only a minor fraction necrosis (Fig. 1c; for gating, see Fig. S3b). Apoptosis induction was confirmed by SubG1 quantification (Fig. 1d), where we observed a significantly higher level of apoptosis in unstimulated vs. stimulated cells following irradiation with 1 Gy (Fig. 1e). The difference in cell death level between stimulated vs. unstimulated cells was already obvious 12 h after irradiation indicating that radiation-induced apoptosis in PBLCs is an early event (Fig. 1c and Supplement Fig. S3e for 72 h posttreatment values). Overall, the data revealed that, contrary to our expectation, unstimulated lymphocytes are more radiosensitive than stimulated lymphocytes.

**Fig. 2:**
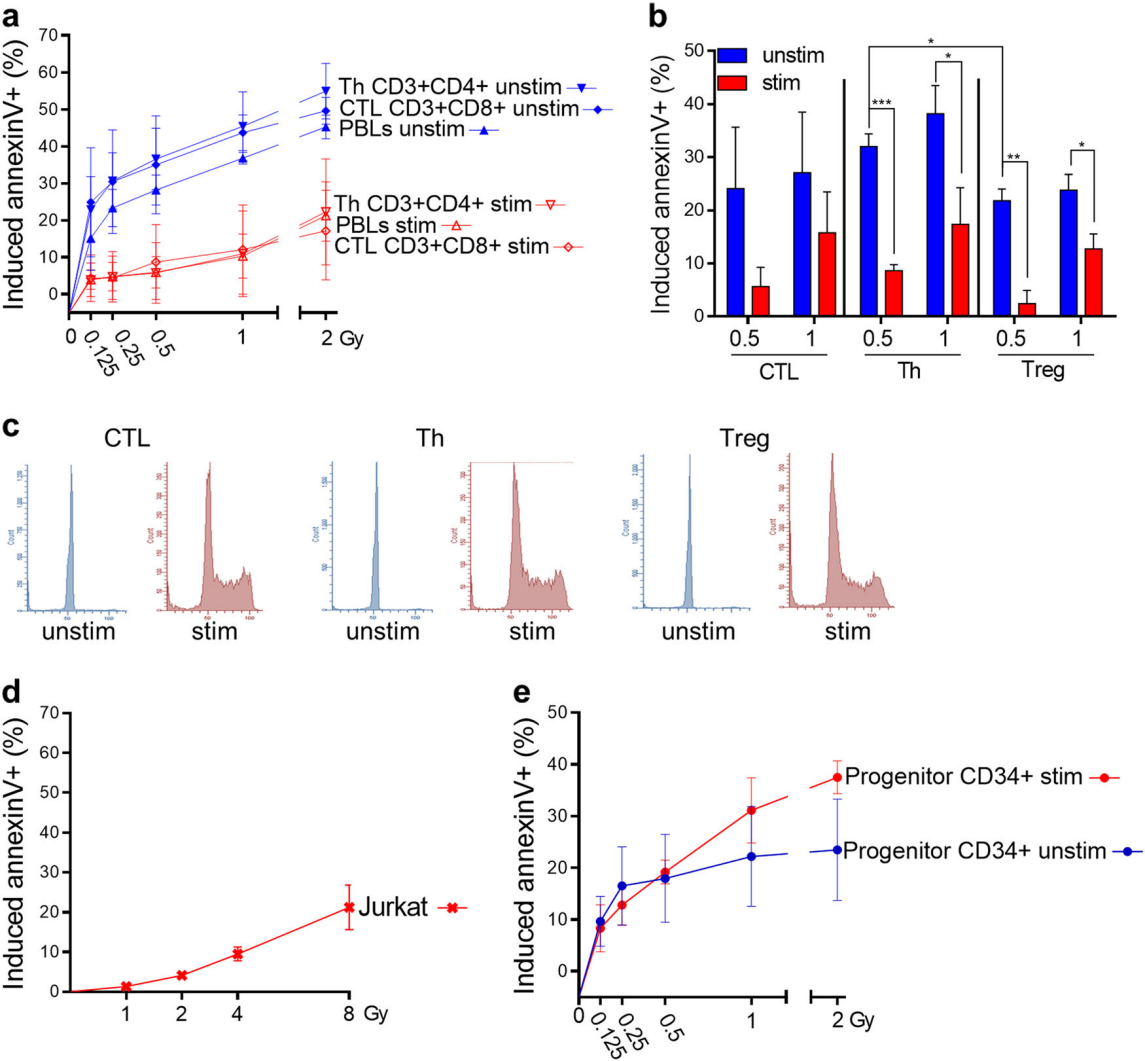
Radiation response of unstimulated vs. stimulated T-cell subtypes and CD34-positive progenitor cells. **a** CD3/CD28 stimulation protects PBLCs as well as CD3+CD4+ Th and CD3+CD8+ CTL within PBLCs after low (0.125 and 0.25 Gy) and high (0.5, 1, and 2 Gy) doses of IR. Cell death (annexinV+) was determined 24 h after irradiation (flow cytometry, *n* = 3, mean value, SD, *t*-test *p < 0.05 significant for PBLCs unstim vs. stim at 0.25, 0.5, 1, and 2 Gy; Th unstim vs. stim at 0,125, 0.25, 0.5, 1, and 2 Gy; CTL unstim vs. stim at 0.25, 1, and 2 Gy). Gatings are described in Figure S4a. **b** A radioprotective effect resulting from CD3/CD28 stimulation was also observed for magnetic bead-isolated CTL, Th, and Treg (annexinV staining 72 h after 0.5 and 1 Gy IR, *n* = 3, *t*-test, *p < 0.05, **p < 0.01). **c** Cell cycle distributions of CD3/CD28-stimulated and unstimulated magnetic bead-isolated CTL, Th and Treg Reihenfolge CTL, Th and Treg. **d** Induced death of Jurkat cells 48 h after different doses up to 8 Gy of IR. Jurkat cells display a very high radioresistance (*n* > 4). **e** Stimulation of CD34-positive progenitor cells with a specific cytokine expanding cocktail showed no protection towards IR (cell death measured by annexinV staining 24 h after IR with flow cytometry, *n* = 3, mean value, SD). Cell cycle distributions of 3 days cytokine-treated and -non-treated CD34-positive progenitor cells are shown in Figure S4b

PBLCs contain a heterogeneous T-cell population comprising CTLs, Th cells, and regulatory T cells (Treg). In the unstimulated status, these populations are slightly different in their radiation sensitivity, as revealed by their dose–response curves (Fig. 2a; see also Supplement Fig. S4a for representative blots). Following stimulation of these T-cell populations with CD3/CD28, they all became clearly more radioresistant (Fig. 2a). This was confirmed using freshly purified CTL, Th, and Treg from the peripheral blood. Stimulation of the affinity-purified populations immediately after collection resulted in a clear decrease in their radiation-induced level of apoptosis (Fig. 2b). Flow cytograms confirmed that non-stimulated CTL, Th, and Treg were arrested in G0/G1, whereas upon stimulation they started proliferation and were randomly distributed in the cell cycle (Fig. 2c).

Jurkat cells, which are immortalized human leukemia T cells, do replicate similar to stimulated PBLCs. Interestingly, Jurkat cells are highly radioresistant. Irradiated with 2 Gy they hardly die and even at a dose of 8 Gy they do not undergo significant apoptosis/necrosis (Fig. 2d). Thus, they are similar to stimulated lymphocytes in their radioresistance. We also measured the radiation response of unstimulated and stimulated CD34 hematopoietic progenitor cells, which were isolated by magnetic bead separation from peripheral blood. The CD34 status was routinely checked by flow cytometry (see Supplement Fig. S2). The cells were arrested in G0 and start proliferation following incubation in stem cell expansion medium (Supplement Fig. S4b). Apoptosis (annexinV + ) was induced already with a radiation dose of 0.125 Gy (Fig. 2e), pointing to the high sensitivity of CD34 + progenitor cells to radiation. Surprisingly, contrary to CTL, Th, and Treg cells, CD34 + cells did not become radioresistant if they were cultured in expansion medium and thus stimulated to proliferation.

**Fig. 3:**
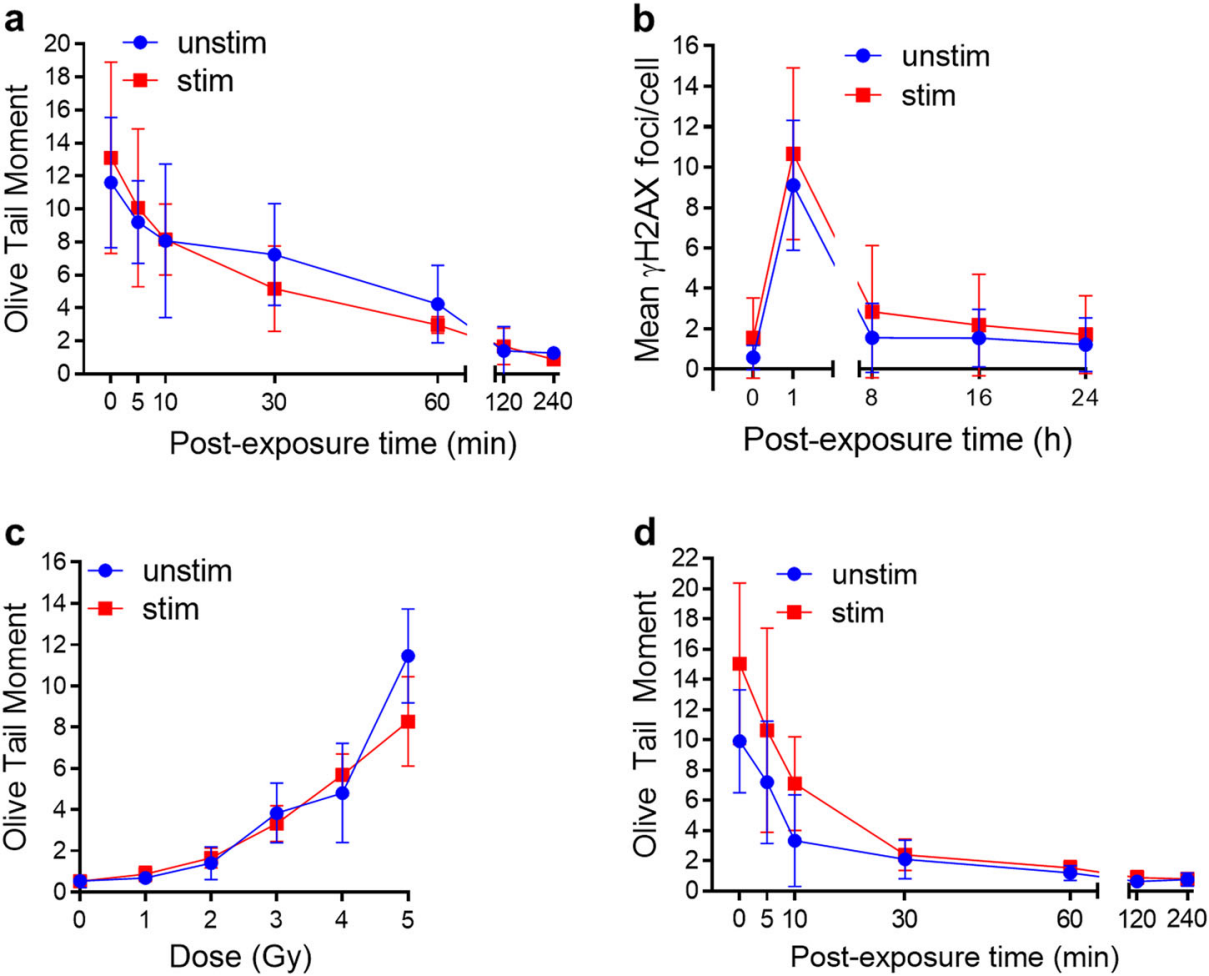
Radiation-induced DNA damage and its repair in unstimulated compared with stimulated PBLCs. **a** DNA double-strand break repair kinetics determined by neutral comet assay at different time points directly (0 min) and up to 240 min after 5 Gy (*n* = 3, 50 cells per sample, mean value, SD). **b** γH2AX-kinetic in non-irradiated (0 h) and 1 Gy-irradiated PBLCs 1–24 h post treatment. **c** DNA single-strand breaks 1 min after different doses of IR were analyzed by the alkaline comet assay (*n* = 3, 50 cells per sample, mean value, SD). **d** DNA single-strand break repair kinetics determined by alkaline comet assay at different time points directly (0 min) and up to 240 min after 5 Gy (*n* = 3, 50 cells per sample, mean value, SD). Representative pictures of neutral and alkaline comets are available in Figure S6a, b

As DSB is the principal toxic lesion induced by IR, we studied DSB formation by the neutral comet assay in non-proliferating and proliferating PBLCs. Interestingly, no differences in the induction and repair kinetics were observed (Fig. 3a and Supplement Fig. S6a for representative stainings). This was confirmed by staining of γH2AX foci, which was similar 60 min after irradiation with 1 Gy (Fig. 3b). The DSB repair kinetic determined through the γH2AX assay was also similar in non-stimulated and stimulated PBLCs (Fig. 3b).

The majority of lesions induced by IR are SSBs and base damage, which can be detected by the alkaline comet assay. As shown in Fig. 3c, the induction of SSB and alkali-labile sites is similar in unstimulated and stimulated PBLCs. The same was true for the repair of these lesions, which was nearly complete 120 min after irradiation (Fig. 3d and Supplement Fig. S6b for representative pictures). Overall, the data indicate that resting and proliferating lymphocytes do not differ in their efficiency in repairing IR-induced DNA breaks.

**Fig. 4:**
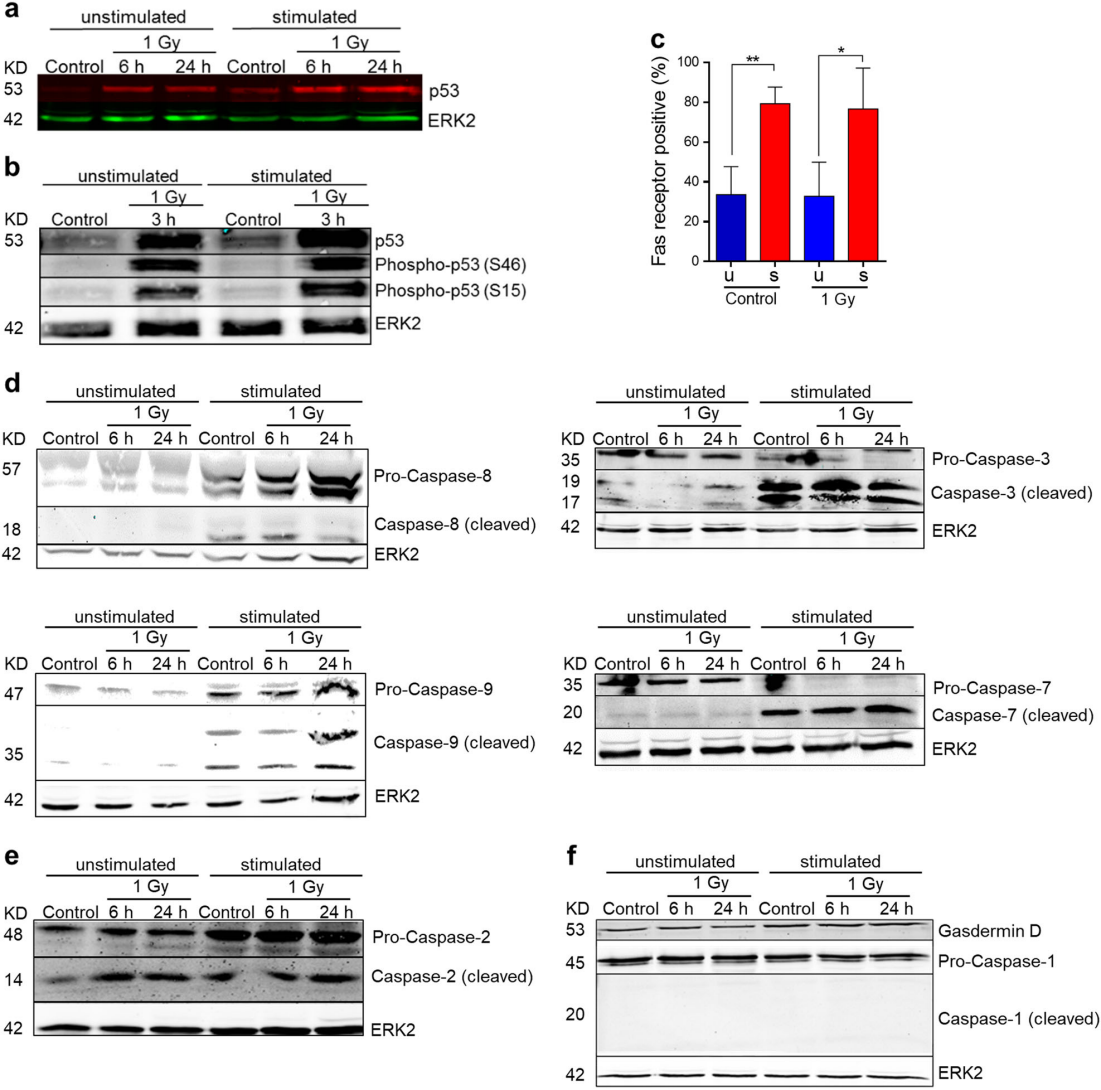
Induction of p53 and cleavage of caspases in unstimulated and stimulated PBLCs after 1 Gy irradiation (western blottings). **a**, **b** Expression and phosphorylation of p53 at Serin 46 and Serin 15. There were no differences in radiation-induced p53 between unstimulated and stimulated PBLC detectable. However, p53 expression was slightly induced upon CD3/CD28 stimulation (control, stim PBLC) (*n* = 3). **c** Quantification of Fas receptor in non-irradiated and irradiated PBLCs (*n* = 3, *t*-test *p < 0.05, **p < 0.01). Stimulated PBLC showed a significant higher level of Fas expression compared with unstimulated cells, independent from exposure to IR. **d** Expression and cleavage of initiator caspases-8 and -9, and executive caspases-3 and -7 in stimulated PBLCs, control, and following IR (controls) (*n* = 3). **e** Caspase-2 was cleaved in both unstimulated and stimulated PBLCs (*n* = 2). **f** In unstimulated and stimulated PBLCs, no cleavage of caspase-1 was visible. Gasdermin D, a substrat of caspase-1 processed in the cell death pathway named pyroptosis, was not cleaved after exposure to IR (*n* = 3). In all western blottings, ERK2 was used as loading control

DNA damage activates p53, which is involved in DNA repair, cell cycle, and death regulation^41^. A clear increase in p53 protein expression was observed in both unstimulated and stimulated PBLCs at 6 and 24 h post irradiation (Fig. 4a), demonstrating that both are responding by p53 stabilization following DNA damage. Of note, a slight induction in the basal p53 level was already seen in stimulated, non-irradiated PBLCs (Fig. 4a, b), indicating that stimulation per se may cause replication stress in lymphocytes. We also determined the p53 activation level by measuring p53-Ser15 and p53-Ser46. These phosphorylations were strongly induced already 3 h after irradiation and there were obviously no differences between unstimulated and stimulated cells (Fig. 4b). Phosphorylation of p53 at Ser46 is mediated by the kinase HIPK2, which was shown to be a pro-apoptotic event^42^.

p53 is a transcription factor stimulating Fas (CD95/Apo-1) receptor expression, which regulates the extrinsic apoptotic pathway^43^. Fas was detected by means of a fluorochrome-coupled antibody and Fas-positive lymphocytes were quantified 6 and 24 h after 1 Gy treatment by flow cytometry. As shown in Fig. 4c, stimulated lymphocytes expressed more Fas than unstimulated lymphocytes, irrespective of radiation. This might be explained by the higher basal level of p53, which is upregulated if cells were stimulated by CD3/CD28 (Fig. 4a, b). Interestingly, there was no clear radiation-induced increase in the level of Fas expression, neither in unstimulated nor stimulated lymphocytes (Fig. 4c; see also Supplement Fig. S7b and S7c). This was also observed in Th and CTL, which were gated for Fas expression (Supplement Fig. S7d and S7e). Therefore, it seems that radiation-induced p53 activation in PBLCs has no impact on Fas expression. It should be noted, however, that following stimulation, about 80% of cells were already Fas positive (Fig. 4c) and a further increase cannot clearly be detected by this method.

Next, the activation of caspases was assessed. Following IR, cleaved caspase-8, -9, -3, and -7 were detectable in stimulated, but not to the same extent in unstimulated PBLCs (Fig. 4d). It should be noted that the basal level of caspases was higher in stimulated than unstimulated PBLs, indicating that PBLC stimulation by CD3/CD28 induces a stress response that may prime the cells to undergo apoptosis. Caspase-2 was expressed in the uncleaved and cleaved form both in unstimulated and stimulated PBLCs. It was slightly enhanced following IR in unstimulated cells (Fig. 4e). In contrast, caspase-1, described as inflammatory caspase, was uniformly expressed and the cleaved form was not detectable (Fig. 4f). Cleavage of gasdermin D is a process involved in a cell death pathway called pyroptosis^44^. Gasdermin D is a substrate of activated caspase-1. It was not cleaved, irrespective of the proliferation status and IR treatment (Fig. 4f), which is in line with lack of caspase-1 activation.

**Fig. 5:**
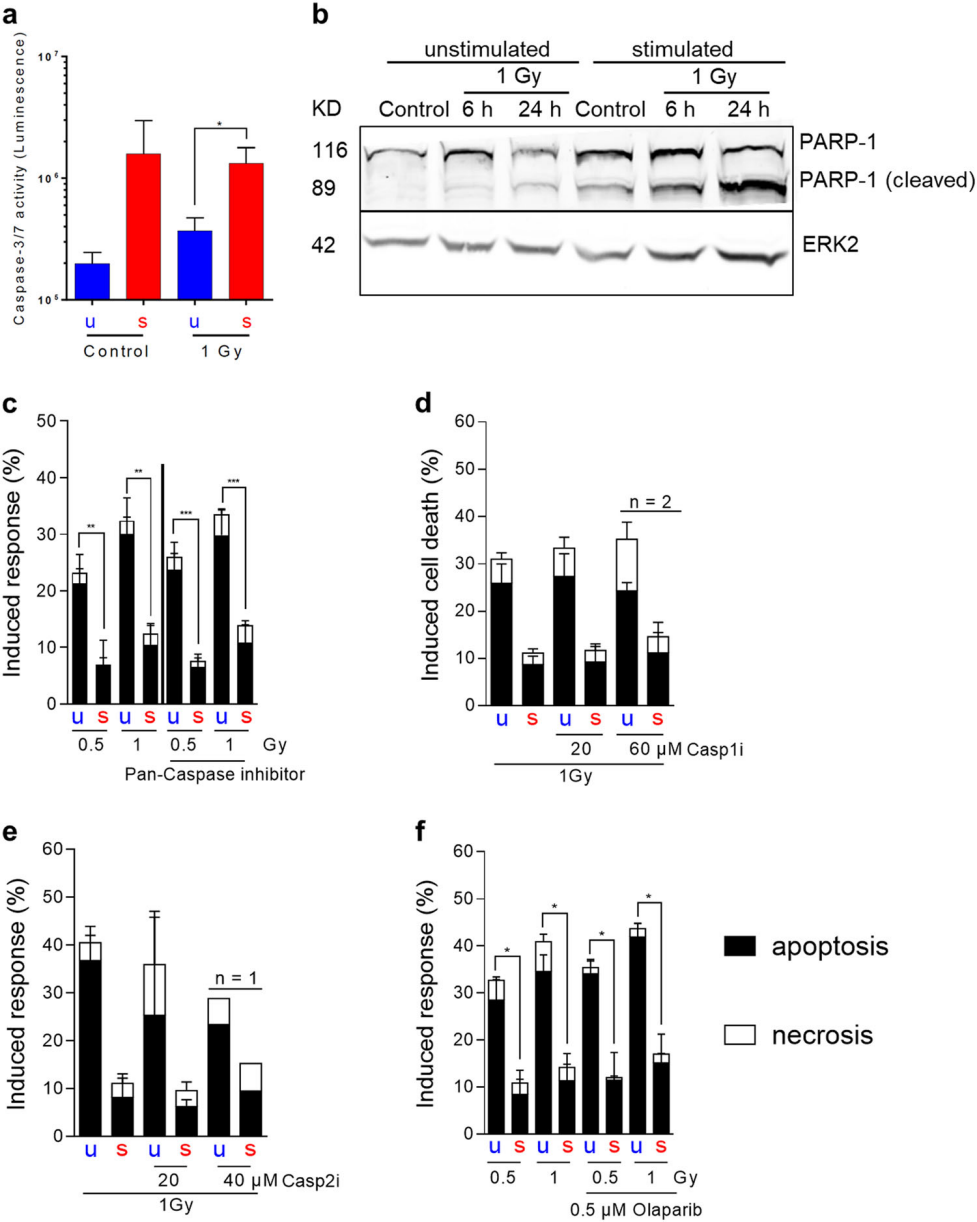
Caspase activity and influence of caspase inhibition on radiation-induced cell death (24 h after IR) in unstimulated and stimulated PBLCs. **a** Stimulated PBLCs showed massive caspase-3/7 activity compared with unstimulated cells, which was independent from treatment with IR. Caspase activity was measured 24 h (Figure S8a for 6 h values) after treatment with IR (*n* = 3, mean value, SD, *t*-test *p < 0.05). **b** PARP-1 cleavage is a specific process induced by activated caspases. Cleavage of PARP-1 could only be determined in stimulated PBLCs (another representative western blot is shown in Figure S8b). **c** Treating PBLCs prior IR with a general caspase inhibitor (20 μM pan-caspase inhibitor Z-VAD-FMK) showed no influence on radiation-induced cell death in unstimulated and stimulated PBLCs. The graphs showed the radiation response without and with inhibitor treatment (*n* = 4, mean value, SD, *t*-test **p < 0.01, ***p < 0.001). The efficiency of the pan-caspase inhibitor was confirmed by a caspase activity assay (Figure S8c). **d**, **e** Inhibition of caspase-2 or caspase-1 showed no effect on radiation-induced cell death (*n* = 2–4, mean value, SD). **f** PBLCs were treated before and after irradiation with olaparib, a specific PARP inhibitor. There was no effect on radiation-induced cell death (*n* = 3, mean value, SEM, *t*-test *p < 0.05)

To substantiate the data, the activity of the executioner caspases-3 and -7 was measured 24 h after IR. Similiar to Fas, a higher caspase-3/-7 activity was found in stimulated compared with unstimulated cells (Fig. 5a), which supports the western blot data shown in Fig. 4d. Caspase-3/-7 activity was not enhanced following IR (Fig. 5a and Supplement Fig. S8a for 6 h after IR), which is again in line with the activated caspase protein level (Fig. 4d). We also studied PARP-1 cleavage, a well-known substrate of caspase-3/7. PARP-1 was also expressed at higher level in stimulated cells and there was a further increase 6 and 24 h after IR (Fig. 5b and Supplement Fig. S8b). Thus, surprisingly, the stimulated lymphocytes displayed PARP-1 cleavage following irradiation, although these cells proved to be radiation resistant.

In view of this data, we addressed the question of whether caspase inhibition exerts an effect on radiation sensitivity of lymphocytes. Unstimulated and stimulated PBLCs were treated or not with a pan-caspase inhibitor 60 min before irradiation and analyzed by annexinV/PI staining after 24 h (for the efficiency of the inhibitor, see Supplement Fig. S8c). No differences in IR-induced cell death were found between pan-caspase inhibitor-treated and non-treated PBLCs (Fig. 5c), indicating that caspase inhibition does not protect lymphocytes from IR-induced cell death. In addition, the effect of inhibition of caspase-1 and caspase-2 on IR-induced cell death was determined. Again, inhibition of either one of these caspases had no significant impact on IR-induced cell death in both unstimulated and stimulated lymphocytes (Fig. 5d for caspase-1 and Fig. 5e for caspase-2 inhibition).

As we observed a difference in the cleavage status of PARP-1 in unstimulated and stimulated cells (Fig. 5b), we studied the influence of the PARP-1 inhibitor olaparib on IR-induced cell death (see Supplement for testing the inhibitor efficiency Fig. S9a). Treatment with olaparib affected the radiation response neither of unstimulated nor stimulated PBLC (Fig. 5f and Supplement Fig. S9b for a higher concentration of olaparib), indicating that PARP-1 is not involved in cell death induction in PBLCs following IR, which is in accordance to the low level of necrosis induced in lymphocytes by IR. In line with this, inhibition of RIPK1, which is involved in the process of necroptosis^45^, had also no effect on IR-induced cell death (see data shown in Fig. 7a). Apoptosis-inducing factor (AIF) translocation from mitochondria into the nucleus is a PAR-dependent process in the parthanatos cell death pathway^46^. Immunohistochemistry of AIF revealed there is no translocation of AIF into the nucleus (Supplement Fig. S9c), which may indicate that parthanatos is not a key event in IR-induced PBLC killing.

**Fig. 6:**
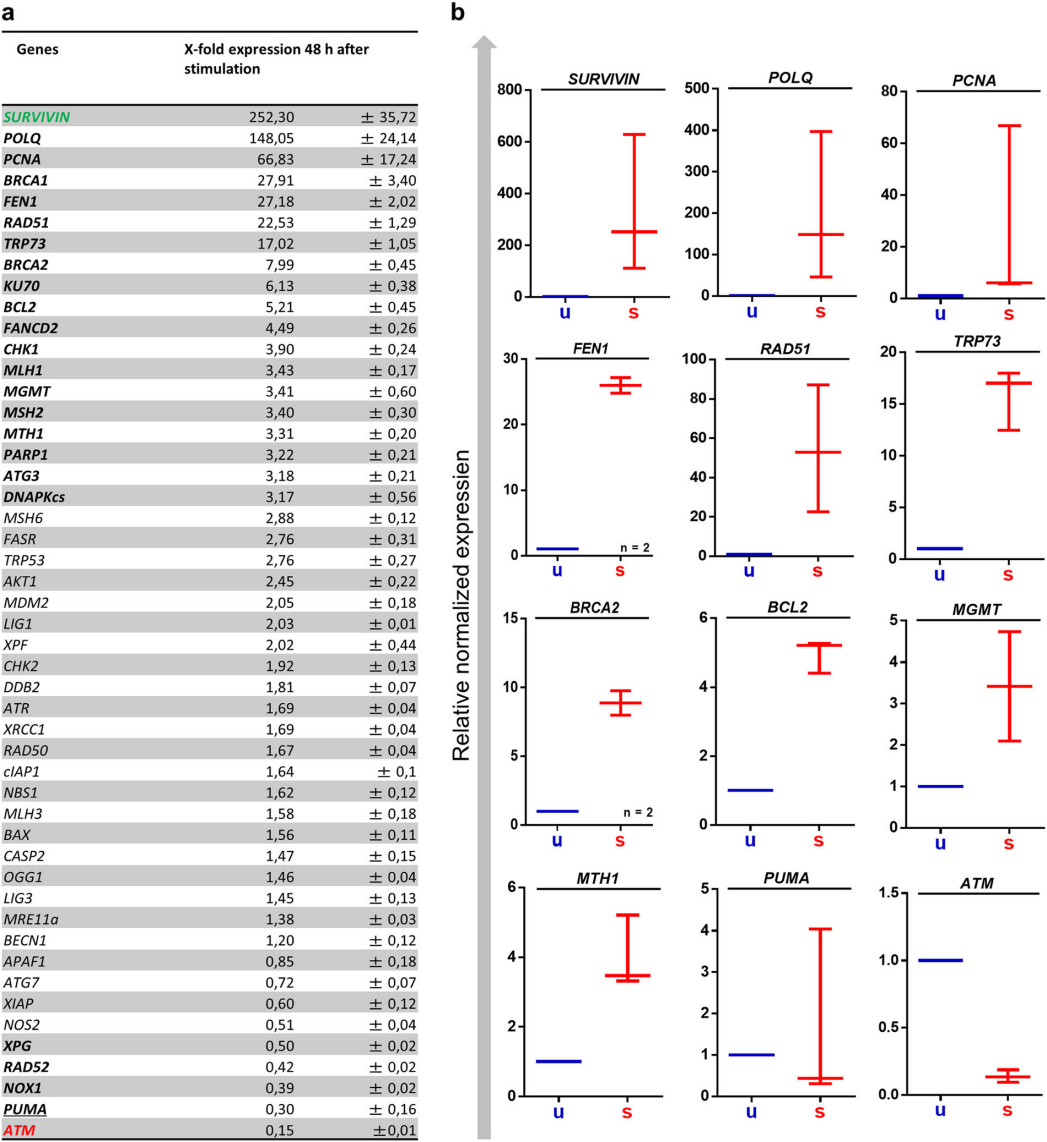
Expression of DNA repair and DNA damage response genes in CD3/CD28-stimulated PBLCs in comparison with unstimulated PBLC. **a** Following T-cell receptor activation, most genes involved in DNA repair and DNA damage response became upregulated. ATM belongs to the group of genes that were downregulated following CD3/CD28 stimulation. Relative expression levels were normalized to unstimulated PBLCs, whose expression level was set to 1. **b** Expression analysis of pooled data from freshly isolated lymphocytes of three independent donors. ATM displayed consistent downregulation, irrespective of donor

To gain better insight into the signaling mechanism in PBLCs following IR, we measured the expression of critical stress response genes in unstimulated vs. stimulated cells. The quantification revealed that the majority of genotoxic stress response genes were upregulated following CD3/CD28 stimulation (Fig. 6a). There was, however, one striking exception, namely ATM, which is the major DNA damage response kinase activated by DSB. ATM was well expressed in unstimulated PBLCs and was found to be significantly downregulated following PBLC stimulation (Fig. 6b).

**Fig. 7:**
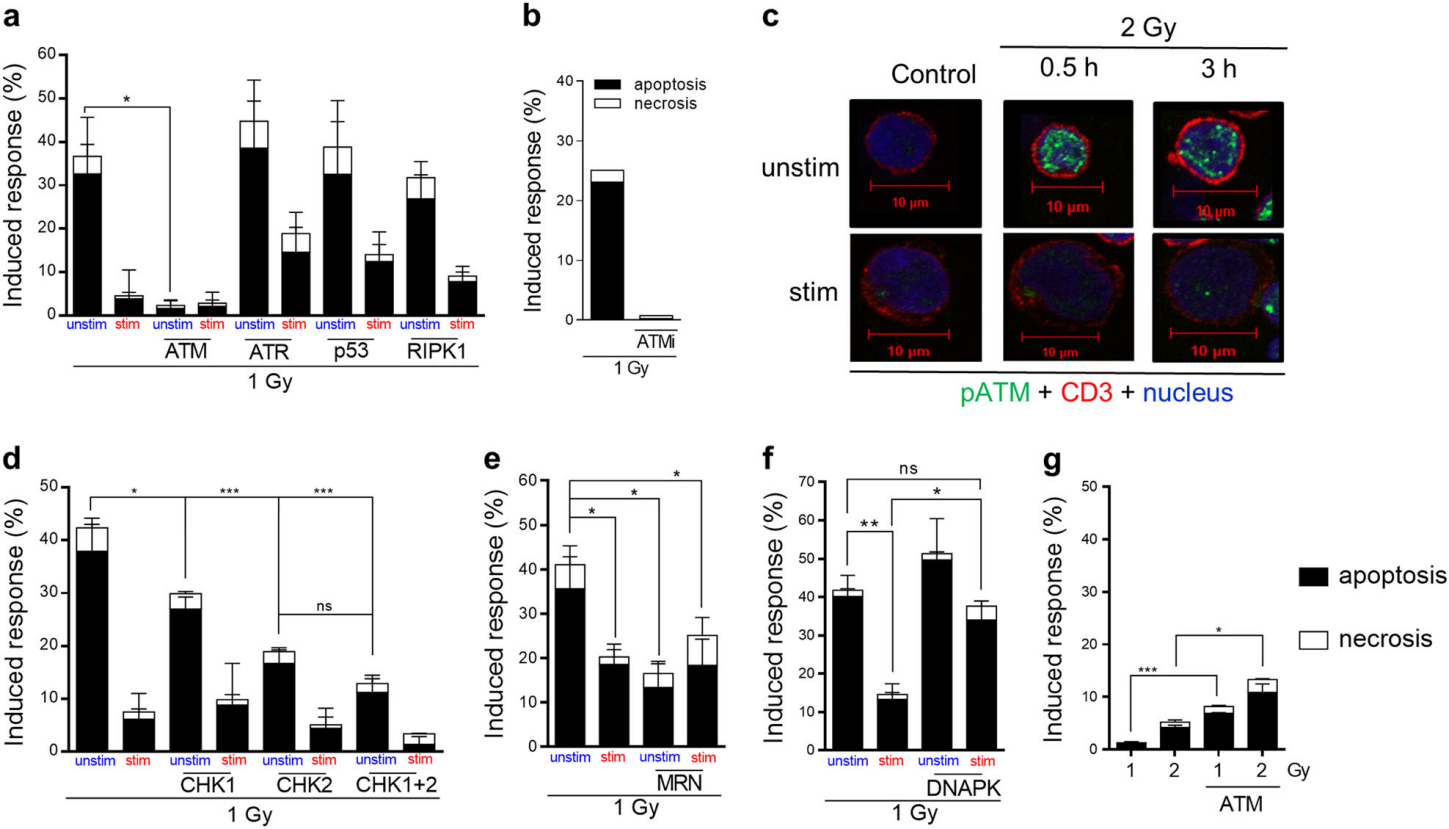
Inhibition of DNA damage response proteins as ATM, CHK1, CHK2, and MRE 11 (MRN complex) protects PBLCs from irradiation-induced cell death (measured 24 h after IR). **a** Inhibition of ATM prior irradiation with 1 Gy significantly protects unstimulated PBLCs from radiation-induced cell death (see Fig. S10a with a lower ATM inhibitor concentration (3 µM) and DMSO control). An inhibition of ATR, p53, and RIPK1 (a marker of necroptosis) had no protective effect on radiation-induced cell death (Fig. S10c for a higher p53 inhibitor concentration) (*n* = 3, mean value, SD, *t*-test *p < 0.05). **b** The alternative ATM inhibitor KU-55933 (10 µM) markedly reduced cell death in unstimulated PBLCs. **c** Representative pictures of phospho-ATM (green) immunostaining revealed higher signals after irradiation in unstimulated compared with stimulated CD3 (red) T cells (blue nucleus) (Fig. S10d for quantification). **d** Inhibition of ATM downstream factors CHK1 and CHK2 protect unstimulated PBLCs from irradiation-induced cell death (*n* = 3–5, mean value, SD, two-way ANOVA (Tukey), *p < 0.05, ***p < 0.001). **e** Inhibition of the ATM upstream factor MRN resulted in radioprotection of unstimulated lymphocytes. **f** However, inhibition of the DNA damage sensor DNA-PK sensitized unstimulated and stimulated PBLCs (**e**, **f**, *n* = 3, mean value, SD, two-way ANOVA (Tukey), *p < 0.05, **p < 0.01). The descriptions of the inhibitors are listed in Materials and Methods. **g** Cell death induction 48 h after 1 and 2 Gy IR in ATM-inhibited Jurkat cells. Inhibition of ATM significantly sensitizes Jurkat cells to IR (*n* = 3–5, mean value, SD, *t*-test, *p < 0.05, ***p < 0.001)

The downregulation of ATM prompted us to determine the effect of inhibitors of the DNA damage response pathway on radiation-induced apoptosis in PBLCs, using pharmacological inhibitors of ATM, ATR, and p53. The data revealed that inhibition of ATM significantly protected unstimulated lymphocytes from IR-induced death (Fig. 7a and Fig. S10a; for testing the efficiency of the ATM inhibitor, see Supplement Fig. S10b). Data were confirmed using an alternative ATM inhibitor (Fig. 7b). Inhibition of p53 showed no significant effect on IR-induced cell death in unstimulated lymphocytes (data are shown in Supplement Fig. S10c).

We also checked the phosphorylation level of ATM in irradiated unstimulated and stimulated lymphocytes. Unstimulated lymphocytes displayed clearly a higher level of phosphorylated ATM compared with stimulated cells as measured 30 min and 3 h after irradiation (Fig. 7c for representative staining; for quantification, see Supplement Fig. S10d). Furthermore, we assessed whether inhibition of specific ATM targets, notably CHK, had an impact on PBLC killing. Inhibition of CHK2, the major ATM target, significantly reduced IR-induced cell death in unstimulated, but not stimulated PBLCs (Fig. 7d). There was also an effect of CHK1 inhibition, but CHK2 was more effective than CHK1 inhibition regarding protection against radiation-induced death of PBLCs. Inhibition of CHK1 together with CHK2 further reduced the killing response, which was however not significant compared with CHK2 alone. Similar to ATM, inhibition of the ATM upstream factor and DNA damage sensor MRE11 (part of the trimeric MRN complex) also caused radioprotection in unstimulated lymphocytes, while stimulated cells remained unaffected (Fig. 7e). Interestingly, inhibition of DNA-PK showed significant radiosensitization, which was most obvious in stimulated lymphocytes (Fig. 7f). Taken together, the data revealed that ATM as well was MRN, CHK1, and CHK2 have a clear pro-apoptotic role in IR-induced cell death in unstimulated PBLCs. Stimulation of PBLC results in significant transcriptional downregulation of ATM (Fig. 6) and a lower apoptotic response of stimulated, proliferating cells. Of note, proliferating leukemic Jurkat cells, used as control showed radiosensitization upon ATM inhibition (Fig. 7g), implying that ATM has different roles in IR-induced cell death in cancer vs. primary cells.

**Fig. 8:**
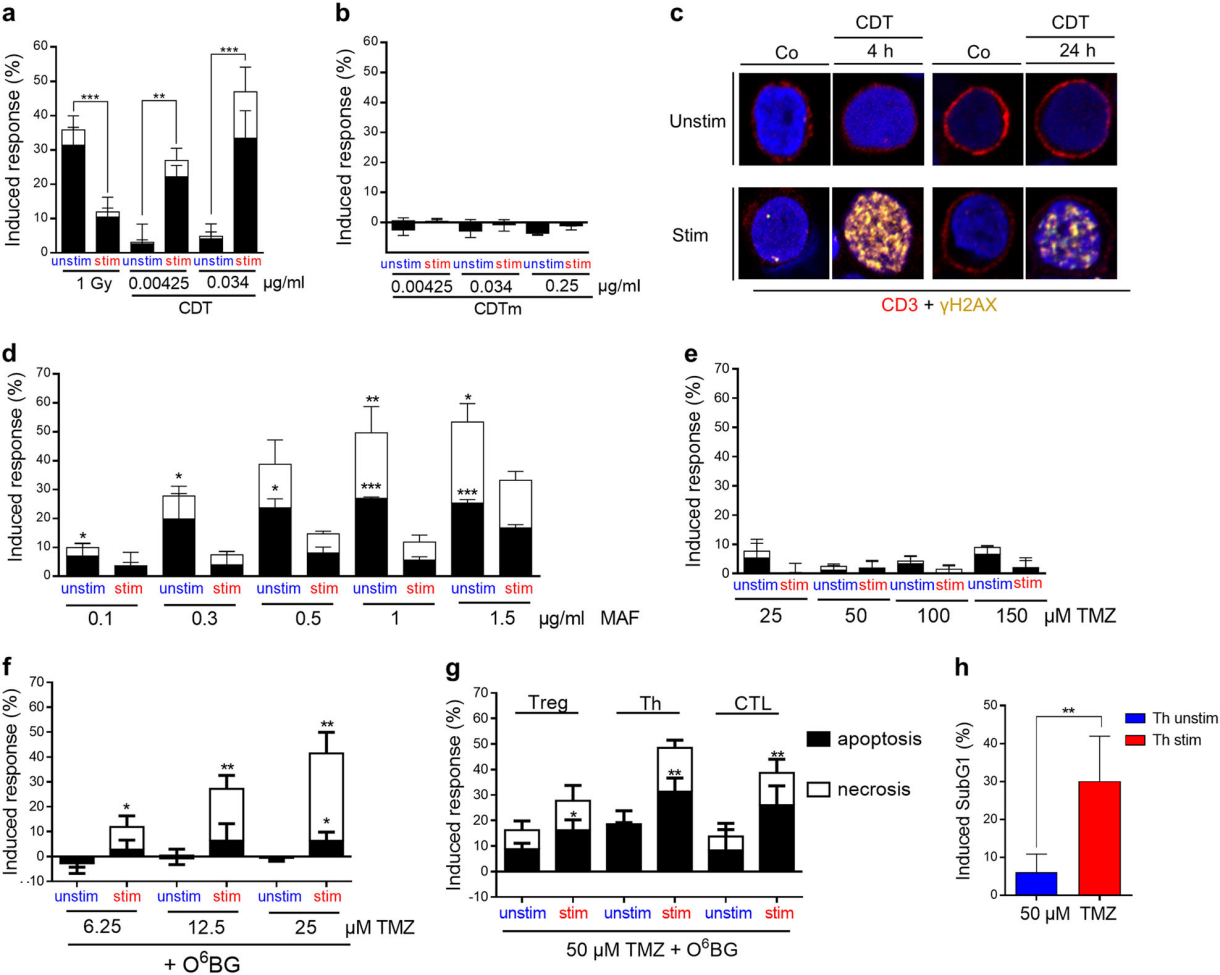
Sensitivity of unstimulated vs. stimulated PBLCs to CDT, MAF, and TMZ. **a** CDT treatment induced significantly more cell death in stimulated than unstimulated PBLCs after 24 h. **b** CDTm, which is mutated in the enzymatic active CDT B-unit, induced neither cell death induction in unstimulated nor stimulated PBLCs. (**a**, **b**, *n* = 3–5, mean value, SD, *t*-test **p < 0.01, ***p < 0.001). **c** Representative pictures of γH2AX (green) staining in unstimulated and stimulated CD3 (red) T cells within PBLC. CDT induced massive DNA damage in stimulated compared with unstimulated cells (quantification of γH2AX intensity is shown in Fig. S11a). **d** Mafosfamide treatment induced after 24 h significantly more cell death (apoptosis and clearly necrosis) in unstimulated than stimulated PBLCs (*n* = 3, mean value, SD, *t*-test, *p < 0.05, **p < 0.01, ***p < 0.001). **e** Without MGMT inhibition, TMZ did not induce significant cell death ( < 10 %) in PBLCs. Apoptosis and necrosis were measured after 72 h. **f** MGMT depletion by pre-treatment with O^6^BG increased significantly TMZ-induced cell death in stimulated cells, already at very low doses of 6.25 and 25 µM TMZ (*n* = 4, mean value, SD, *t*-test, *p < 0.05, **p < 0.01). **g** The sensitizing effect of CD3/CD28 stimulation towards TMZ was also obvious in magnetic bead-isolated and MGMT depleted Treg, Th, and CTL (cell death was measured 72 h after TMZ treatment, *n* = 5, mean value, SD, *t*-test, **p < 0.01, ***p < 0.001). **h** Apoptosis was measured in unstimulated and stimulated Th by SubG1 quantification

To study whether the high sensitivity of resting human PBLCs is a specific radiation response or pertains to other DNA-damaging agents as well, we compared unstimulated and stimulated PBLCs following treatment with different genotoxins such as CDT, mafosfamide (MAF), and TMZ. CDT is a radiomimetic bacterial toxin, which induces DNA SSBs and DSBs. Treatment of CD3/CD28-stimulated and unstimulated PBLCs with CDT resulted in dose-dependent increase in apoptosis and, partly necrosis, which was observed only in stimulated PBLs. This is completely contrary to what was shown for IR (see Fig. 8a for comparison with IR). A mutant DNase I-deficient CDT was completely ineffective in inducing apoptosis and necrosis in PBLCs (Fig. 8b), showing that DNA strand break formation is required for cell death induction. Treatment with active CDT induced γH2AX foci in stimulated PBLCs already after 4 h, which was almost lacking in unstimulated cells (Fig. 8c; for quantification, see Supplement Fig. S11a). Thus, the response to CDT of unstimulated and stimulated PBLCs was essentially different compared with IR.

Treatment of unstimulated and stimulated PBLs with MAF, an active derivate of the crosslinking agent cyclophosphamide that is often used in cancer therapy^33,47^, resulted in more cell death (apoptosis and necrosis) in unstimulated compared with stimulated PBLCs (Fig. 8d). This resembles the response following IR. Of note, in contrast to IR, a higher fraction of necrotic cells was observed (∼50% necrosis), indicating agent-specific cell death response.

TMZ is a methylating agent first line used in malignant glioma therapy. It is also applied in refractory metastatic melanoma therapy. The main toxic adduct is *O*^6^-methylguanine (*O*^6^MeG), which is repaired by the suicide enzyme O6-methylguanine-DNA methyltransferase (MGMT)^8,48^. Without the MGMT inhibitor *O*^6^-benzylguanine (*O*^6^BG), unstimulated and stimulated PBLCs displayed nonsignificant cell death following TMZ treatment (Fig. 8e). However, depleting MGMT with *O*^6^BG before TMZ resulted in significant death, which was observed exclusively in stimulated PBLCs (Fig. 8f). The data were confirmed by comparing the subpopulations Treg, Th, and CTL. As shown in Fig. 8g, similar responses were observed upon TMZ treatment in these T-cell populations. In all cases, the stimulated T cells were more TMZ sensitive. Similar data were obtained when apoptosis was quantified by subG1 flow cytometry (Fig. 8h for Th and Supplement Fig. S11b for Treg and CTL). These experiments demonstrate that stimulated PBLCs are equipped with mechanisms that trigger cell death and provide evidence for a remarkable agent specificity in eliciting the cytotoxic response following DNA damage in unstimulated vs. stimulated blood lymphocytes.

## Discussion

The majority of lymphocytes in the peripheral blood is in the resting state. Following infection, however, a subset of lymphocytes, notably the T-cell population, becomes activated by cytokines, starts proliferation, and gives rise to an expanded T-cell pool. Key mediators in this scenario are cytokines activating CD3 and CD28 receptors of T cells. Activated T cells have a role not only in the defense against infections but also in the host response against malignantly transformed cells^6^. As cancer cells are usually treated with IR and/or chemotherapeutics, the question arises on how activated and proliferating T cells compared with non-activated cells respond to genotoxic insults. In this study we addressed this question. We show that human proliferating T cells are clearly more resistant to IR than their non-proliferating counterpart. This finding was surprising and not according to our initial expectation, as there is a general believe that proliferating cells, including cancer cells, are more sensitive to genotoxic insults than resting cells of the same origin.

We should stress the point that non-proliferating lymphocytes are highly sensitive to IR, undergoing cell death already with a radiation dose as low as 0.2 Gy, at which adherent normal^49^ and tumor cell lines^50^ do not show yet cytotoxic effects. Apoptosis induction in PBLCs and T-cell subpopulations below doses of 2 Gy were observed following annexinV staining and confirmed by subG1 flow cytometry (Fig. 2a and Supplement Fig. S3 and S4). The high-radiation sensitivity of non-proliferating lymphocytes is in line with a previous report on phytohemagglutinine (PHA)-stimulated lymphocytes, which showed less IR-induced death in stimulated than unstimulated lymphocytes^51^. Of note, PHA is a quite unspecific lymphocyte stimulator. Here we extended this observation, showing that increase in radioresistance pertains to the total T-cell population stimulated through CD3/CD28 receptor activation and the T-cell subpopulations CTL, Th, and Treg. Intriguingly, resting CD34 + progenitor cells isolated from peripheral blood were sensitive to IR and did not show protection if they were cultivated in expansion medium. This indicates that the high-radiation sensitivity of resting T cells is an inherent property of this cell type.

Lymphocytes undergo death upon radiation exposure mainly through apoptosis; necrosis was observed to a much less extent (e.g., 48 h after 1 Gy, 38% annexinV +/PI −, indicating early and late apoptosis, and 10% annexinV −/PI +, indicating necrosis). In contrast to this is the finding that upon IR resting PBLCs did not show significant caspase activation and PARP-1 cleavage and posttreatment with caspase inhibitors had no effect on the radiation-induced level of apoptosis, which indicates that radiation-induced cell death in PBLCs is caspase independent. Interestingly, stimulation of T cells with CD3/CD28 alone gave rise to increase in Fas receptor expression and massive caspase activation and PARP-1 cleavage, and radiation exposure did not further aggravate this response. This is in line with a report showing that T-cell receptor activation itself results in high caspase activity, which is thought to be a physiological process of activated T cells^52,53^. Caspase cleavage after T-cell receptor stimulation was also described as a result of increased granzyme B level, which specifically cleaves caspase-3^54^. The high caspase level following CD3/CD28 activation is in strong contrast to the lower apoptotic response of these cells to IR. Another mechanism could be pyroptosis, which designates a caspase-3-independent programed death mechanism. It rests on caspase-1 activation and gasdermin D cleavage. However, we could neither observe caspase-1 nor gasdermin D cleavage in irradiated resting PBLCs. We also found no evidence for necroptosis, as cells were mostly PI negative and inhibition of RIPK1, a mediator of necroptosis^44–46^, did not rescue T cells from IR-induced cell death.

Irrespective of the executive mechanism involved, the high-radiation response of unstimulated lymphocytes requires explanation. Both unstimulated and stimulated lymphocytes repair IR-induced DNA damage with similar efficiency, as shown by the alkaline comet and γ-H2AX foci assay measuring DNA SSBs and DSBs, respectively. A significant difference was observed, however, in the DDR gene expression pattern. Following CD3/CD28 activation, most genes involved in DNA repair and DDR became upregulated. There was only one clear exception: ATM, which was expressed at high level in unstimulated lymphocytes and downregulated in CD3/CD28-stimulated lymphocytes. This was observed on RNA level and on the level of the phosphorylated ATM protein. Therefore, it is obvious that the same amount of DNA damage is able to trigger a strong DDR resulting in programmed death in non-stimulated PBLCs compared with the stimulated counterpart. If ATM is causally involved, it was anticipated that inhibition of the ATM pathway in non-stimulated PBLCs abrogates their high-radiation sensitivity. This was indeed the case. Pharmacological inhibitors targeting ATM, MRE11 (the component of MRN complex) (Fig. 7e), and CHK1 and CHK2 significantly reduced the death level of irradiated unstimulated PBLCs. This is a remarkable effect, as in most experimental systems ATM is a pro-survival factor and, therefore, ATM inhibitors are used as radio-sensitizer, which was shown, e.g., for glioblastoma cells^55^. Interestingly, as shown here, in proliferating T-cell leukemia Jurkat cells, ATM inhibition exerted also a radio-sensitizing effect, which is in stark contrast to non-transformed T cells. Inhibition of DNA-PK_CS_ sensitized both non-stimulated and stimulated PBLCs, indicating that DNA-PK has a pro-survival role irrespective of the activation status of T cells, likely to be through their role in non-homologous end-joining. Although p53 is downstream of ATM, we did not observe clear differences in the p53 stabilization, and the p-p53(Ser46) and p-p53(Ser15) level following IR treatment. Therefore, we suppose that ATM in non-stimulated PBLCs results in activation of not yet identified target(s) that are causally linked to a caspase-independent death pathway, which is characterized by DNA fragmentation, subG1, and annexinV positivity. A candidate might be c-Abl, which was shown to trigger pro-apoptotic signaling after ATM activation via mitochondrial damage activation^50,56^. We should note that CD3/CD28 stimulation of PBLCs itself resulted in an increase of the p53 protein level, which might indicate that T-cell receptor stimulation results in replicative stress activating the canonical DDR pathway. Taken together, following CD3/CD28 treatment, stimulation occurred on the level of Fas receptor and caspase activation, which however resulted only in a pre-apoptotic state without executing the final steps. Moreover, the data revealed that ATM is a key pro-death factor in non-stimulated T cells.

T cells in the peripheral blood have a long life span (of about 2 years) and, therefore, it is pertinent to speculate that the highly responsive ATM pathway in T cells is required for eliminating cells harboring DNA damage. This prevents from the accumulation of point mutations and gross chromosomal changes, and finally malignant transformation leading to leukemia. In fact, ATM is frequently mutated in lymphoid malignancies^57^, which is consistent with the hypothesis that T cells harboring DNA damage are eliminated in an ATM-dependent manner. As activated T cells are essentially required for the immune response, it is reasonable to posit that the ATM killing pathway becomes silenced in order to protect activated T cells against radiomimetic stress such as reactive oxygen species (ROS), which is produced by activated immune cells such as granulocytes, monocytes, and macrophages. Thus, we observed that non-stimulated T cells co-cultivated with activated, ROS-producing macrophages undergo death at high level^58^. Although it is very likely that CD3/CD28-activated T cells resist macrophage-generated ROS, evidence still needs to be provided.

Our studies on the sensitivity of non-stimulated vs. stimulated PBLCs (each obtained from the same donors) to other genotoxicants revealed that non-stimulated PBLCs are more sensitive than their stimulated counterparts to the cyclophosphamide analog MAF, which is similar to what was found for IR. Previously, we reported that Treg are, at a low-dose level, significantly more sensitive to MAF than Th and CTL because of ineffective removing of interstrand crosslinks (ICLs)^59^. Thus, CD3/CD28 stimulation ameliorated the capacity to remove MAF-induced DNA damage by increasing the DNA crosslink repair efficiency. Our gene expression studies indicate an increased expression of FANCD2 and PCNA upon CD3/CD28 stimulation. Both proteins are involved in DNA ICL repair^60,61^. Clearly, further studies are required to clarify the mechanism of resistance of proliferating PBLCs to crosslinking agents, which is clearly clinically relevant.

It might be argued that T cells upon stimulation are not equipped with functions that are required for triggering apoptotic death. This, however, is not the case, as shown by the response of proliferating T cells to TMZ. This anticancer drug induces a dozen of DNA methylation adducts of which *O*^6^MeG is the main killing lesion, providing that the repair protein MGMT is not expressed or pharmacologically inactivated by *O*^6^BG. Under the condition of MGMT depletion, non-proliferating PBLCs were highly resistant to TMZ, whereas CD3/CD28-stimulated T cells died to a large extent. This supports the model that DSB are produced after erroneous mismatch repair on *O*^6^MeG/thymine lesions, which trigger the apoptotic pathway^48,62^. This process requires DNA replication^63^. The killing effect of TMZ specifically on proliferating T cells and the high-radiation response of non-proliferating T cells shows that T cells, irrespective of their proliferation status, are equipped with apoptosis executing factors.

To gain more insight into the kind of DNA damage triggering apoptosis following IR, we performed experiments with the CDT, which is produced by Gram-negative pathogenic bacteria to modulate host cell functions. This bacterial protein was reported to be a radiomimetic agent in fibroblasts, inducing persistent DNA strand breaks^35,64^. To our surprise, similar to TMZ, CDT was more toxic in stimulated than unstimulated PBLCs. It is important to note that CDT induced a high amount of γH2AX foci only in stimulated T cells. This is in contrast to IR, which induced the same level of γH2AX foci in resting and proliferating T cells. It is therefore pertinent to conclude that DNA SSBs produced by CDT at low doses are converted into DSBs only in replicating cells, exerting an effect there, whereas the DSB level produced by IR (1 Gy produces up to 40 DSBs in adherent cell lines^65^) was sufficiently high, in order to activate the death pathway. In line with this, a high DNA fragmentation level after CDT was detected only in stimulated T cells^66^.

Overall, the data show that the effect of genotoxicants on PBLCs is dependent both on their proliferation status and the type of DNA damage induced. The high-radiation sensitivity of resting PBLCs and the radioresistance of activated T cells might explain the high efficiency in the depletion of blood cells during whole-body radiation (for which usually 2 Gy is used) and, at the same time, the survival of tumor-infiltrated, activated T cells following irradiation of the tumor. The same holds true for MAF and its pro-drug cyclophosphamide, against which activated T cells proved to be resistant. We should note that MAF kills specifically Treg^59^, which is harnessed for stimulation of the immune response by low-dose treatment with cyclophosphamide^67,68^. The inability of TMZ to kill resting T cells might explain why TMZ is a well-tolerated drug^69^, even in a daily and dose-escalation schedule^70^. The finding implicates, however, that in the therapeutic situation the drug is likely to kill activated T cells that infiltrated the tumor tissue in a host-defense reaction and, thereby, the antitumor T-cell response will very likely be attenuated. Therefore, in immunotherapy settings, DC vaccination together with radiotherapy and/or cyclophosphamide treatment for boosting the immune response is expected to enhance the therapeutic index, while DC vaccination together with TMZ treatment is likely to be counter-productive as activated CTLs might be killed selectively.

Overall, this comparative analysis revealed a high sensitivity of resting T cells and gain-of-radiation resistance following their activation, which is mediated by the MRN-ATM-CHK2 pathway triggering caspase-independent apoptosis. This radiation response pathway is likely of utmost importance in eliminating T cells harboring non-repaired DSBs that may lead otherwise to genomic changes and malignant transformation. For genotoxicants inducing primary DNA damage that requires replication to be transformed into killing lesions, proliferating T cells were highly sensitive. This might be harnessed in approaches aimed at attenuating immune responses, e.g., for the treatment of autoimmune diseases.

## Methods

### Cell isolation and culture

Peripheral blood mononuclear cells (PBMCs) were separated by Histopaque (Histopaque 1077, Sigma-Aldrich) density gradient centrifugation from leukocyte-rich plasma named Buffy Coat, which was provided by the blood bank from healthy human donors. CD4 + CD25 + Treg cells, CD4 + Th cells, and CD8 + CTLs were isolated from PBMCs with magnetic bead-coupled antibodies (Miltenyi Biotec) and phenotyped with fluorochrome-coupled antibodies by flow cytometry as described (Supplement Fig. S1)^59^. CD34-positive progenitor cells were isolated from PBMCs with the CD34 MicroBead Kit, UltraPure human (Miltenyi Biotec) (Supplement Fig. S2a and S2b).

PBMCs were cultivated in six-well Corning plates with RPMI and 1.5% autologous serum. Autologous serum was separated in the process of Ficoll (Histopaque 1077) density gradient centrifugation from buffy coat. After 30 min of cultivation (37 °C at 5% CO_2_), monocytes attached to the bottom of the plates, whereas peripheral blood lymphocytes remained in the supernatant. The amount of CD3 + T cells within the PBLC population ranged between 70% and 85% (Supplement Figure S3a).

Jurkat cells (ACC 282, DSMZ) were cultivated in RPMI medium (Gibco Life Technologies) containing 10% fetal calf serum (Biochrome AG). Cells (2.5 × 10^5^) were irradiated and cultivated per well of a 12-well plate for cell death analysis.

### Stimulation of PBLCs and CD34 progenitor cells

Each well of a 24-well plate was coated upon incubation for 1–2 h in an incubator with 4 µl anti-CD3 antibodies (purified NA/LE anti-human CD3, 555329, BD Pharmingen) in 200 µl 0.1 M NaHCO_3_ solution (pH 8.2, sterile). After removing the solution and carefully washing each well with 1 ml phosphate-buffered saline (PBS), 0.5–1 × 10^6^ PBLCs were cultured per well with 1 ml X-VIVO15 containing 10% autologous serum and 2 µl anti-CD28 antibodies (purified NA/LE anti-human CD28, 555725, BD Pharmingen) 48 h for stimulation. Unstimulated cells were cultivated 2 days without CD3/CD28 antibodies. PBLCs were collected and maintained in X-VIVO15 medium without serum for experiments.

CD34-positive progenitor cells, which were isolated by magnetic bead-coupled antibodies, were cultivated for expansion in Stem MACS HSC Expansion Media XF (Miltenyi Biotec) 1:100 with Stem MACS HSC Expansion Cocktail (Miltenyi Biotec) for 6 days, to stimulate cell division. The CD34 marker expression remained preserved upon expansion (Supplement Figure S2c).

### Exposition to γ-irradiation and treatment with genotoxins and pharmacological inhibitors

Cells were transfered into Greiner Tubes and irradiated within a gammacell irradiator 2000 (Cs-137 source, Molsgaard Medical, Denmark) in a dose equivalent time frame. The cells were treated 1 h before γ-irradiation or genotoxicant treatment with the following pharmaceutical inhibitors: ATM inhibitor KU-55933 (stock 10 mM, end 10 µM), ATM inhibitor KU-60019 (stock 3 mM, end 3 µM), ATR inhibitor VE-822 stock 10 mM, end 10 µM), DNA-PK inhibitor KU-0060648 (stock 0.9 mM, end 5 µM) (Selleckchem, Munich), caspase-2 inhibitor Z-VDVADFMK (stock 2 mM, end 20 µM), caspase-1/ICE inhibitor Z-WEHDFMK (stock 2 mM, end 20 µM), pan-caspase inhibitor Z-VADFMK (stock 20 mM, end 20 µM) (R&D Systems, Inc., Minneapolis, USA), CHK1 inhibitor UCN-01 (stock 1 mM, end 150 nM), CHK2 inhibitor II (stock 10 mM, end 10 µM) (Sigma-Aldrich St. Louis, Germany), cyclic pifithrin-alpha (stock 30 mM, end 30 µM) (Biomol, Cayman Chemical, Ann Arbor, USA), mirin (stock 25 mM) (Tocris Bioscience, Bristol, UK), necrostatin-1 (stock 50 mM, end 100 µM) (Cayman Chemical, Ann Arbor, USA), and olaparib (stock 1 mM, end 0.5 µM) (Absource Diagnostic, Munich). Following genotoxins were directly added to the cultured cells: recombinant CDT (stock 400 ng/μl) and mutant CDT lacking DNase I activity (stock 400 ng/μl) expressed and purified as described^71^, MAF (stock 1 mg/ml) (ASTA Medica, Frankfurt, Germany), and TMZ (stock 35 mM) (Schering-Plough, USA).

### Quantification of cell death

Cell death was measured by annexinV/PI and SubG1 staining. Cells were transferred to 15 ml Greiner tubes and centrifuged 10 min at 1200 r.p.m. After removing of the supernatant, the pellet was resuspended in 25 µl 1 × annexinV-binding buffer (10 × annexinV-binding buffer; pH 7.4, 10 mM HEPES, 140 mM NaCl, 2.5 mM CaCl_2_, 0.1 % bovine serum albumin) with 1.25 µl annexinV–fluorescein isothiocyanate (FITC) (Miltenyi Biotec) and incubated for 20 min in the dark at room temperature. AnnexinV-binding buffer (220 µl) and 5 µl PI were added to the solution, stored on ice and annexinV, and PI-positive cells were determined by flow cytometry (BD FACSCanto) as shown in Supplement Figure S3b. Cell death of Th was determined by staining PBLCs with CD3-PE + CD4-VioBlue + annexinV-APC, CTL with CD3-PE + CD8-APC + annexinV–FITC with appropriate compensation steps (representative blots are shown in Supplement Figure S4). The induced effect of irradiation or genotoxic treatment on cell death was calculated by subtracting the basal level of the untreated control or inhibitor-treated sample from the irradiated respectively genotoxin treated sample. For SubG1 staining, 80% of ice-cold ethanol was added to the cell pellets, which were stored for 1 h up to 2 week at − 20 °C. The suspension was centrifuged at 4 °C for 10 min at 1200 r.p.m. Supernatant was removed and pellet resuspended in 333 µl PBS + 1 µl RNase (stock 10 mg/ml). After 1 h at room temperature, 164 µl PI (stock 50 µg/ml) was added to the solution and samples were stored at ice until DNA fragmentation was determined by flow cytometry. Data analyzes was performed by FACSDiva software and flowing software 2. SubG1 staining was also used to calculate cell proliferation. Fas receptor was stained with CD95-eFluor®450 (Affymetrix eBioscience) in combination with CD3-PE and CD4-VioBlue, respectively; CD8-APC antibodies (Miltenyi Biotec) were analyzed by flow cytometry. As isotype control for CD95, a specific mouse IgG1 Isotype Control eFluor®450 was used (Affymetrix eBioscience).

### Single-cell gel electrophoresis

DNA SSBs and DSBs were analyzed by the neutral and alkaline comet assay^72^. PBLCs in 120 µl low-melting agarose were transferred on 1.5% agarose-coated slides. After 5 min of storage at 4 °C, slides were incubated 1 h at 4 °C with the neutral lysis buffer (2.5 M NaCl, 100 mM EDTA, 10 mM Tris, 1 % sodium lauroyl sarcosinate, 1% Triton X-100, 10% dimethyl sulfoxide (DMSO), pH 7.5) or 50 min with the alkaline lysis buffer (2.5 M NaCl, 100 mM EDTA, 10 mM Tris, 1% sodium lauroyl sarcosinate, 10% DMSO, 1% Triton X-100, pH 10). Electrophoresis was performed for 22 min at 25 V in 4 °C cold electrophoresis buffer (90 mM Tris, 90 mM boric acid, 2 mM EDTA, pH 7.5) or 15 min at 23 V (300 mM NaOH, 1 mM EDTA, pH > 13). Samples were washed in ddH_2_O, fixed in 98% ethanol and air-dried for 2 h. After staining with PI, DNA migration was analyzed by fluorescence microscopy and the fluorescence intensity quantified using Kinetic Imaging 4.0.2 software (Optilas, Puchheim). Per sample, the mean olive tail moment of 50 cells was determined.

### Whole-cell extracts and western blot analysis

Cell pellets were resuspended in 50–200 µl lysis buffer (50 mM Tris, pH 7.5, 250 mM NaCl, 1 mM EDTA, 0.1% Triton X-100) + 1 × proteinase inhibitor cocktail complete (mini EDTA-free, Roche) and incubated for 30 min on ice. Alternatively, cells were resuspended on ice with RIPA buffer (50 mM Tris pH 8, 150 mM NaCl, 1 mM EDTA, 1% NP-40, 0.5 deoxycholic acid, 0.1% SDS, 0.1% sodium azide, 100 mM phenylmethylsulfonyl fluoride, 200 mM Na_3_VO_4_, 1 M dithiothreitol, 1 × proteinase inhibitor). Samples were centrifuged at 4 °C with 14,000 r.p.m. and pellets stored at − 20 °C. Protein concentrations were measured by the Bradford method. Cell extracts were heated for 5 min at 95 °C or 56 °C. Cell extracts were separated on a 10% or 12% SDS-polyacrylamide gel at 120 V and blotted onto a nitrocellulose membrane for 1 h at 300 mA using a buffer composed of 100 ml 5 × Laemmli buffer (30 g Tris, 144 g lycine in 1 l ddH_2_O), 200 ml Methanol, 10 ml 10% SDS, and 1000 ml ddH2O. The following antibodies were used: caspase-3, pAB from rabbit (35, 19, 17 kDa), caspase-7, pAB rabbit (35, 20 kDa), caspase-8, mAB mouse (57, 48, 18 kDa), caspase-9, pAB rabbit (47, 37, 35 kDa), caspase-2, mAB mouse (48, 14, 12 kDa), p53, mAB mouse, phospho-p53_Ser15_, pAB rabbit (all from Cell Signaling Technology), phospho-p53_Ser146_, pAB rabbit, ERK2 (42 kDa) (both Santa Cruz Biotechnology), PARP-1, mAB mouse (116, 89 kDa) (kind gift from Professor A. Bürkle, Konstanz), caspase-1, mAB rabbit (30–45 kDa, 22 and 20 kDa), gasdermin D, mAB mouse (53 kDa), XRCC1, pAB rabbit (70 kDa) (all from Abcam), HIPK2, pAB rabbit (100 kDa) (kind gift from Professor T. Hofmann, Heidelberg, Mainz). Protein detection was performed by the Odyssey imaging system (LI-COR Biosciences) with secondary antibodies coupled to infrared dyes (IRDye 800CW and IRDye 680).

### Caspase-3/7 activity assay

Caspase activity was measured with the Caspase-Glo® 3/7 assay (Promega, Heidelberg). Ten thousand cells/well were cultured in white 96-well plates, incubated with the reaction mix and 1 h later the luminescence signal was measured by the TriStar 2 Multimode Reader LB942 (Berthold Technologies). Blank (with medium only) was subtracted from values.

### Detection of reactive oxygen species

ROS were measured with the dye CM-H_2_DCFDA (Invitrogen). The cells were incubated with 5 µl CM-H_2_DCFDA (final concentration 6.25 µM) per ml X-VIVO15 medium for 30 min at 37 °C. Cells were washed 2 × with PBS and the fluorescence signal measured by flow cytometry.

### Immunocytochemistry

Cells were transferred on cover slides and fixed for 6 min with ice-cold methanol:acetone (7:3) and afterwards 10 min with 2.5% paraformaldehyde (PFA). Samples were blocked with 10% normal goat serum in PBS. Co-staining of the CD3 T-cell receptor (anti-CD3, mAB rat, MCA 1477, AbD Serotec) with phospho-ATM (anti-phospho S1981 ATM, mAB rabbit, ab81292, Abcam) or γH2AX (anti-phospho S139 Histone γH2A.X, mAB mouse, JBW 301, Merck Millipore; anti-phospho S139 Histone γH2A.X, mAB rabbit, 81299, ChIP Grade, Abcam) within PBLCs was essentially performed as described previously^73^. Secondary antibodies were goat anti-rabbit IgG (H + L) conjugated with Cy3 (112165143, Jackson Immuno Research) and F(ab‘2)-goat anti-mouse IgG (H + L) conjugated with AlexaFluor®488 (A11017, Life Technologies) or F(ab‘2)-goat anti-rabbit IgG (H + L) conjugated with AlexaFluor®488 (A11070, Life Technologies). TO-PRO-3 was used to visualize the nuclei. Slides were covered with Vectashield and fluorescence measured by a confocal Laser Scanning Microscope (LSM 710, Carl Zeiss). For γH2AX single staining, cells were pre-fixed 10 min with 4% PFA and 10 min with 0.5% Triton X-100 in PBS. γH2AX foci were determined by the Metafer imaging system (Carl Zeiss, Göttingen). Cells were fixed for 15 min with 4% PFA and 5 min with ice-cold acetone prior AIF-staining (anti-AIF XP®, mAB rabbit, Cell Signaling). Staining of Poly (ADP-ribose) (PAR) (10 H, mAB mouse, kind gift of Professor Alexander Bürkle, Konstanz^74^) was carried out after fixation and permeabilization of cells for 7 min with ice-cold methanol and incubation with 5% milk powder and 0.1% Tween-20 in PBS. AIF and PAR signals were analyzed by LSM.

### Real-time PCR

RNA was isolated with the NucleoSpin-RNA II Kit (Machery Nagel) as described by the user’s manual. RNA concentration and purity was measured with NanoDrop®-ND-1000 (Nanodrop Technologies). cDNA was generated with the Verso cDNA Kit (AbGene). One microgram RNA was diluted with 11 µl dH_2_O (Ampuwa®, Fresenius) and heated for 5 min at 70 °C in a thermocycler. Nine microliters of Reverse Transcriptase Mix was added (4 µl 5 × cDNA Synthesis buffer, 2 µl dNTP Mix, 1 µl Random Hexamer, 1 µl RT Enhancer, 1 µl Verso Enzyme Mix) and samples heated in a Thermocycler 60 min at 42 °C and 2 min at 95 °C. dH_2_O was added to the samples to reach an end volume of 50 µl. Reverse transcriptase-PCR (RT-PCR) was performed with the following mastermix (SensiMix SYBR + Fluorescein Kit, Bioline): 10 µl 2 × SensiMix SYBR & Fluorescein, 0.5 µl forward primer and 0.5 µl reverse prime, 5.4 µl dH_2_O, 1.6 µl MgCl_2_. Sequences of primers are listed in Supplement Figure S5. Two microliters of cDNA was added per well (96 PCR plate (Bio-Rad) to 18 µl of SYBR-Mastermix. Non-Template-Control contains dH_2_O instead of cDNA; Non-Reverse-Transkriptase-Controls are cDNA samples generated with dH_2_O instead of Verso Enzyme Mix. ERCC6, UBE4A, and ENOX2 (Primeronly geNorm 12 gene kit, Primerdesign) were used as reference genes and defined as suitable with Best Keeper® software. RT-PCR was performed at a C1000 Thermal Cycler CFX96 (Bio-Rad) with the following program: 10 min at 95 °C and 44 cycles with 15 s at 95 °C, 15 s at 56 °C, and 20 s at 72 °C. Melting curve was created by the steps 1 min at 95 °C, 1 min at 55 °C, and 55 °C to 95 °C in 0.5 °C/10 s steps. Gene expression analysis was performed using the Bio-Rad CFX-Manager Software 3.1.

## Electronic supplementary material

Sensitivity of stimulated vs. non-stimulated lymphocytes to ionizing radiation and genotoxic anticancer drugs: key role of ATM in the differential radiation response
