# Supplementary material for: Sensitivity of CD3/CD28-stimulated versus non-stimulated lymphocytes to ionizing radiation and genotoxic anticancer drugs: key role of ATM in the differential radiation response
Source: Cell Death Dis. 2018 Oct 15;9(11):1053. doi: 10.1038/s41419-018-1095-7 (PMC6189042; doi:10.1038/s41419-018-1095-7)
Supplement: Supplementary file 1 — Sensitivity of stimulated vs. non-stimulated lymphocytes to ionizing radiation and genotoxic anticancer drugs: key role of ATM in the differential radiation response [file 41419_2018_1095_MOESM1_ESM.pdf]

Supplemental Material

Sensitivity of CD3/CD28 stimulated versus non-stimulated lymphocytes to ionizing radiation and genotoxic anticancer drugs: key role of ATM in the differential radiation response

Daniel Heylmann, Jennifer Badura, Huong Becker, Jörg Fahrer and Bernd Kaina*

Institute of Toxicology, University Medical Center, Obere Zahlbacher Straße 67, 55131 Mainz, Germany

* Corresponding author:
Prof. Bernd Kaina, Institute of Toxicology, University Medical Center, Obere Zahlbacher Strasse 67, D-55131 Mainz, Germany; Tel: 0049 6131 17 9217, kaina@uni-mainz.de

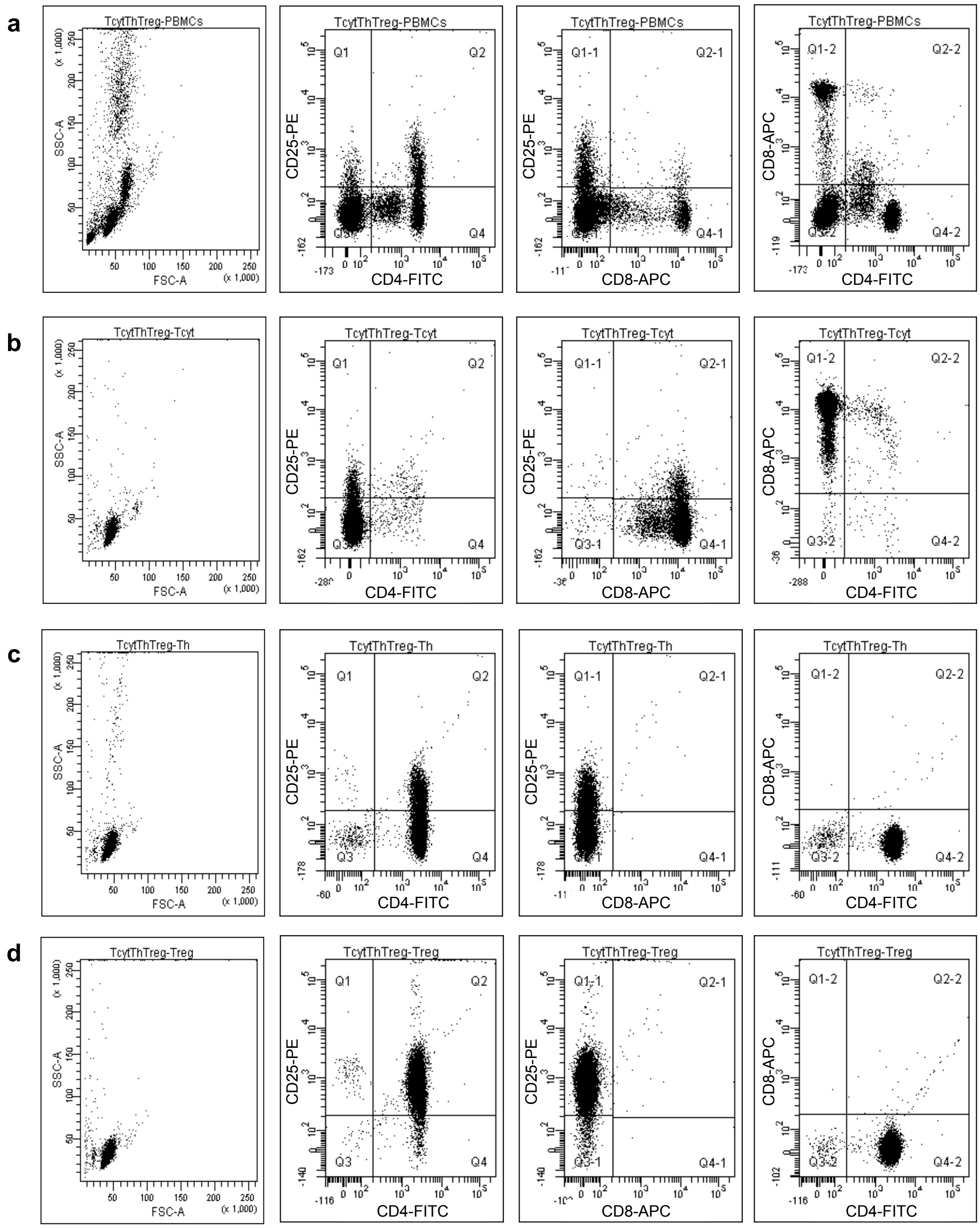

Figure S1 Representative Dot-Blots (flow cytometry) of magnetic bead isolated CTL, Th and Treg from PBMC as described in Heylmann et al. 2013 PLoS ONE 8(12): e83384. (**a**) PBMCs before isolation. (**b**) CTL, (**c**) Th and (**d**) Treg, isolated with anti-CD8, anti-CD4 and Regulatory T cell Isolation Kit from Miltenyi Biotec.

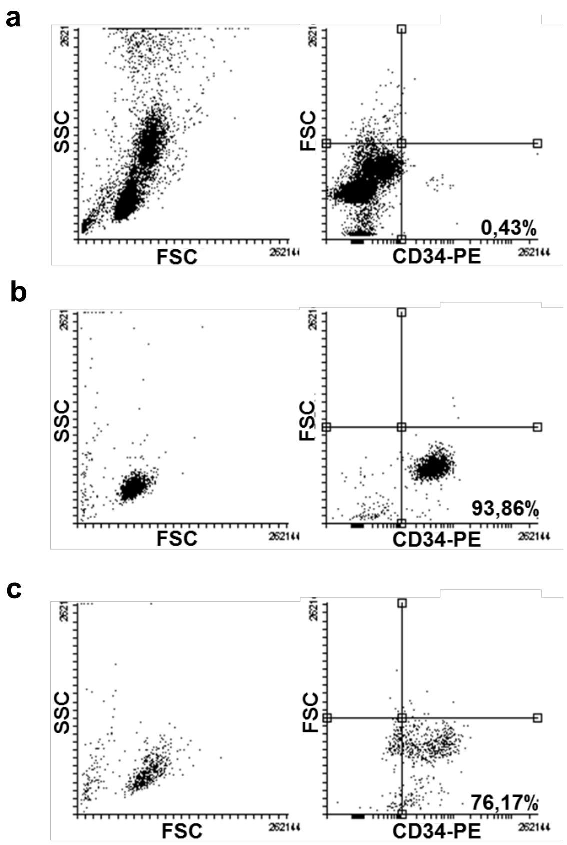

Figure S2 Representative Dot-Blots (flow cytometry) after isolation and expansion of CD34 positive progenitor cells from PBMC. (**a**) PBMC before CD34 isolation. (**b**) Purification of CD34 positive progenitor cells from PBMC with CD34 MicroBead Kit UltraPure from Miltenyi Biotec. (**c**) 6 days expansion of CD34 purified cells with StemMACS HSC Expansion Media XF (130 100 473, Miltenyi Biotec) and StemMACS HSC Expansion Cocktail (130 100 843, Miltenyi Biotec). The CD34 marker remains largely preserved.

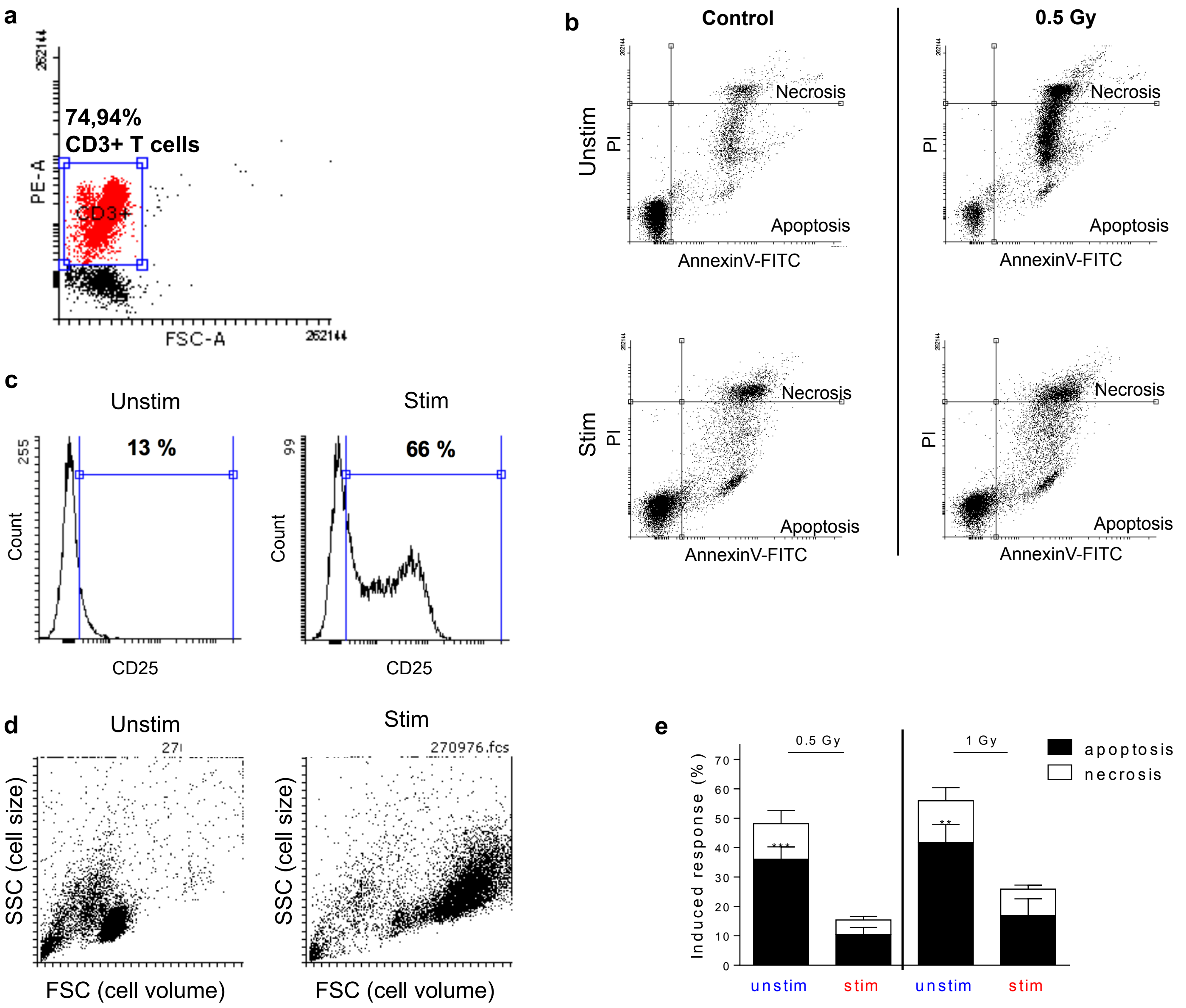

Figure S3 (**a**) CD3-PE staining of T cells within PBLC (flow cytometry). (**b**) Gating of apoptotic (AnnexinV-FITC positive/PI negative) and necrotic cells (AnnexinV-FITC positive/PI positive) within unirradiated (Control) and irradiated (0.5 Gy) unstimulated and stimulated PBLC, analyzed by flow cytometry. (**c**) CD25 staining of unstimulated versus stimulated PBLC. (**d**) Cell size and cell volume (dot blot of sideward- and forward-scatter) of unstimulated and stimulated PBLC, determined by flow cytometry. (**e**) AnnexinV/PI staining (induced cell death) of unstimulated and stimulated PBLC 72 h after 0.5 and 1 Gy (flow cytometry, n = 3 , mean value, SD, t-Test apoptotic part, ** $p < 0.01$).

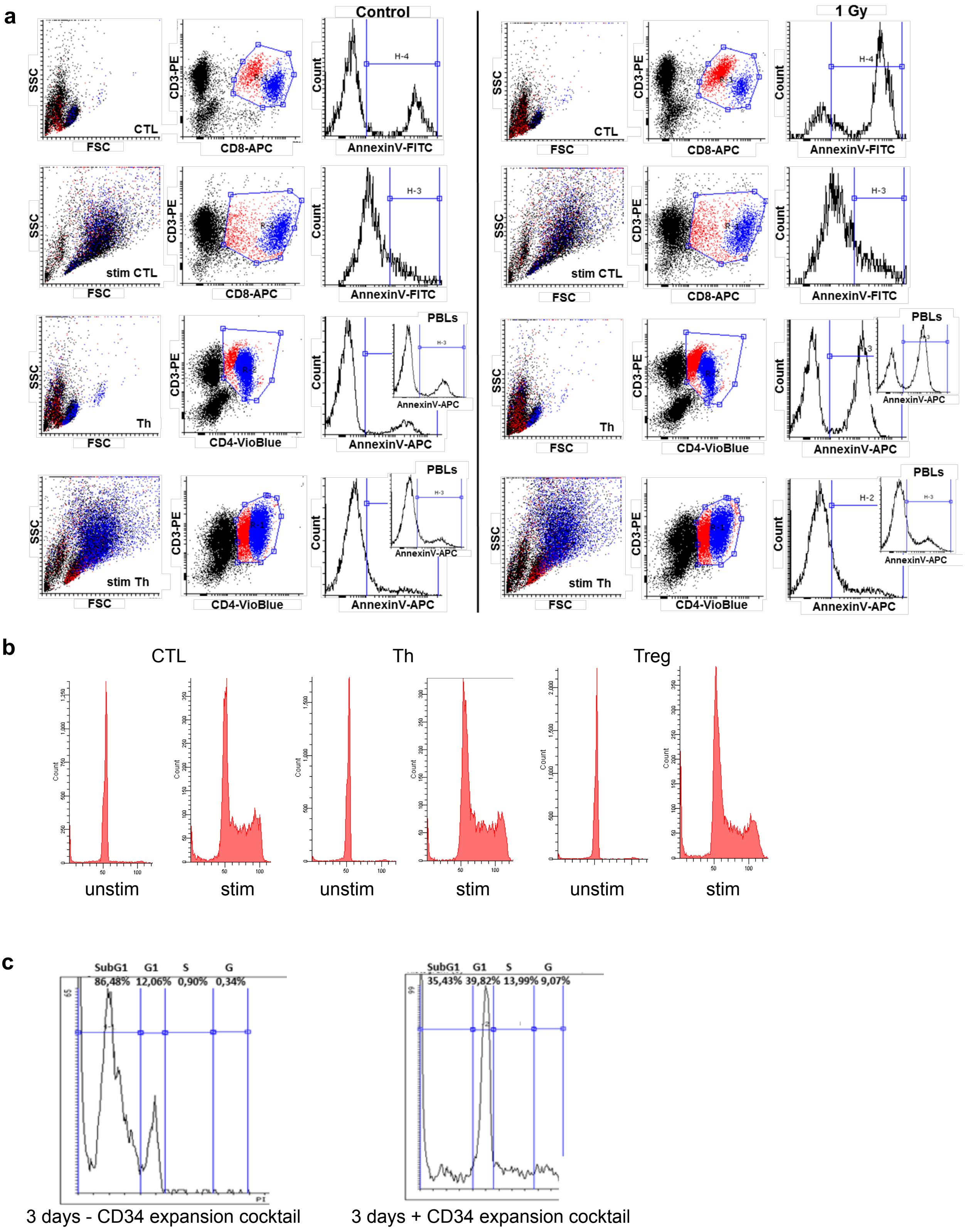

Figure S4 (**a**) Representative gates of AnnexinV-stained CTL (CD3CD8) and Th (CD3CD4) within unstimulated and stimulated PBLC by flow cytometry. (**b**) Cell cycle distributions (SubG1) of CD3-/CD28-stimulated and unstimulated magnetic bead-isolated CTL, Th and Treg. (**c**) Cell cycle distribution (SubG1) of magnetic bead-isolated CD34 progenitor cells 3 days cultivated without and with a specific cytokine expansion cocktail.

GenE	antisense (low)	sense (up)
**SURVIVIN***	ATGACTTGTGTGTGATGA	GTTTGTGCTATTCTGTGAA
**POLQ**	GCAATATCCAATGAAGAGA	ATACTACCTCCAFAFTCT
**PCNA**	AGTGTCACCGTTGAAGAG	TTACCATAGAGATGAATGAACCA
**BRCA1**	CAGTTCCAAGGTTAGAGA	AAGACTTCTACAGAGTGAA
**FEN1**	AACAGCAATCAGGAACTG	CGGGAGAATGACATCAAG
**RAD51***	GGGGTGATCAGTTTCTGTTGC	GCACAATCATCTGCAAGTGGG
**TRP73**	ATCTGGCAGTAGAGTTTC	CACTTTGAGGTCACTTTC
**BRCA2***	CTACACCACCCACCCTTA	CATGACTTGCAGCTTCTCT
**KU70**	AACTATCTCTCTGAATA	TGGGAGTCATATTACAAA
**BCL2**	TCTACTTCCTCTGTGATGT	CGACTCCTGATTCATTGG
**FANCD2**	GGCTGTTACGGAATGAAGA	AGTTGGTGATGGATAAGTTGT
**CHK1**	TAAATCACAATCGCCACTC	CCACCTCTTCATAACAACAA
**MLH1**	CTGAATACCTGCCAACAA	TTACAACATAGCCACGAG
**MGMT***	AATCACTCCGAATTTCAC	CTCTTCACCATCCCGTTT
**MSH2***	CTTCTTCTGGTTCGTCAGTATAGA	ATCATTCTCCTTGGATGCCTTAT
**MTH1**	AAACCAGTAGCTGTCGTCG	CGAAATGCGCCCATGCTG
**PARP1**	CACTTGCTGCTTGTTGAA	GAACGACCTGATCTGGAA
**ATG3**	TTCCAACAATCCACTCTC	AGGACAATATAAGGCTTCAA
**DNAPKcs**	GGATTTCTTCTCTACATTCAC	GCATTACAGACATCTTTAGTT
**MSH6***	GAAATTTAAGCCAGACACTAA	CCTTCACTCTCACTATCC
**FASR**	GGCTTCATTGACACCATT	TTATCTGATGTTGACTTGAGTAA
**TRP53**	ACGGATCTGAAGGGTGAAA	AGCACTAAGCGAGCATG
**AKT1**	TGAAGGTGCCATCATTCT	ATTGTGAAGGAGGGTTGG
**MDM2**	AGGCTATAATCTTCTGAGTC	ATCTTGATGCTGGTGTAA
**LIG1**	CTTTGGAGGTCTTTAGGG	GAAGTGGCAACAGAGAAG
**XPF**	AGATGAATGCTTCTTGACA	GGTTGACTTCTTGACTGATA
**CHK2**	AGGCACCACTTCCAAGAG	GCACTGTCACTAAGCAGAAAT
**DDB2**	GCATTCTGAGATTCCAAAGC	TGTAGCCTGGATGTGTCT
**ATR**	CAGATGACTTCACAGATT	AAGGCAGTTGTATTGAAT
**XRCC1#**	CCTCCTCCTTCACACGGAAC	CTCCCCCTTTGGCTTGAGTT
**RAD50**	TATAAGTTCTCCATTGACATC	AGACGACCATCATTGAAT
**cIAP**	CGATAATACTAGCAATGACCAA	CACCTGGAAACAAAGCATT
**NBS1**	GTCCTGGAGTTGTTGTCTT	ATTGGATTGGCGGTGATT
**MLH3**	CTTCTATTCTCTGCCTAA	TGTAACAGTGTATAACCTA
**BAX**	CAGAAGGCACTAATCAAG	ATCAGATGTGGTCTATAATG
**CASP2**	TCTGGTCACATAGAACAT	GAGAGAAAGAACTGGAATT
**OGG1**	GATGTTGTTGTTGGAGGAA	AAGAGGTGGCTCAGAAAT
**LIG3**	GGAATAGGCACAGTTCTT	GCTATATGTCTTTGGCTTTC
**MRE11a**	GGTTGCCATCTTGATAGTT	GCCTGTCCAGTTTGAAAT
**BECN1**	CTGTGGTAAGTAATGGAG	GGATGGAAGGGTCTAAGA
**APAF1**	AAGACGAAGATTTCCTTA	TGTAGTATCTTTAATTGGTG
**ATG7**	AACCTTGTCCAAGTCTAA	GAACCTTCAGTGAATGTATG
**XIAP**	CCGAAGAGAAACCACATTT	CTGAGCCAGATCAAAGTATG
**NOS2**	CTCCACATTGTTGTTGAT	AATCCAGATAAGTGACATAAG
**XPG**	TCTATCGCTTGAGGATTATGA	CAGTTCAGAAGAGGAAGATG
**RAD52**	TGTTGTATCTTGCCTCCT	CAGTTGCCTCTTGAAGTG
**NOX1**	CACACTTGCGAGGATGAC	ATGACAATAGCCTTGATTCTCA
**PUMA**	TTCAGTTTCTCATTGTTAC	TAAGGATGGAAAGTGTAG
**ATM**	GGAATCTGAATGCCGATCTAG	TATCTGCTGCCGTCAACTA

Figure S5 Primer Sequences. Sigma *Life Science* Array_HD and Array_HUMAN, Eurofins Genomics*, Invitrogen#

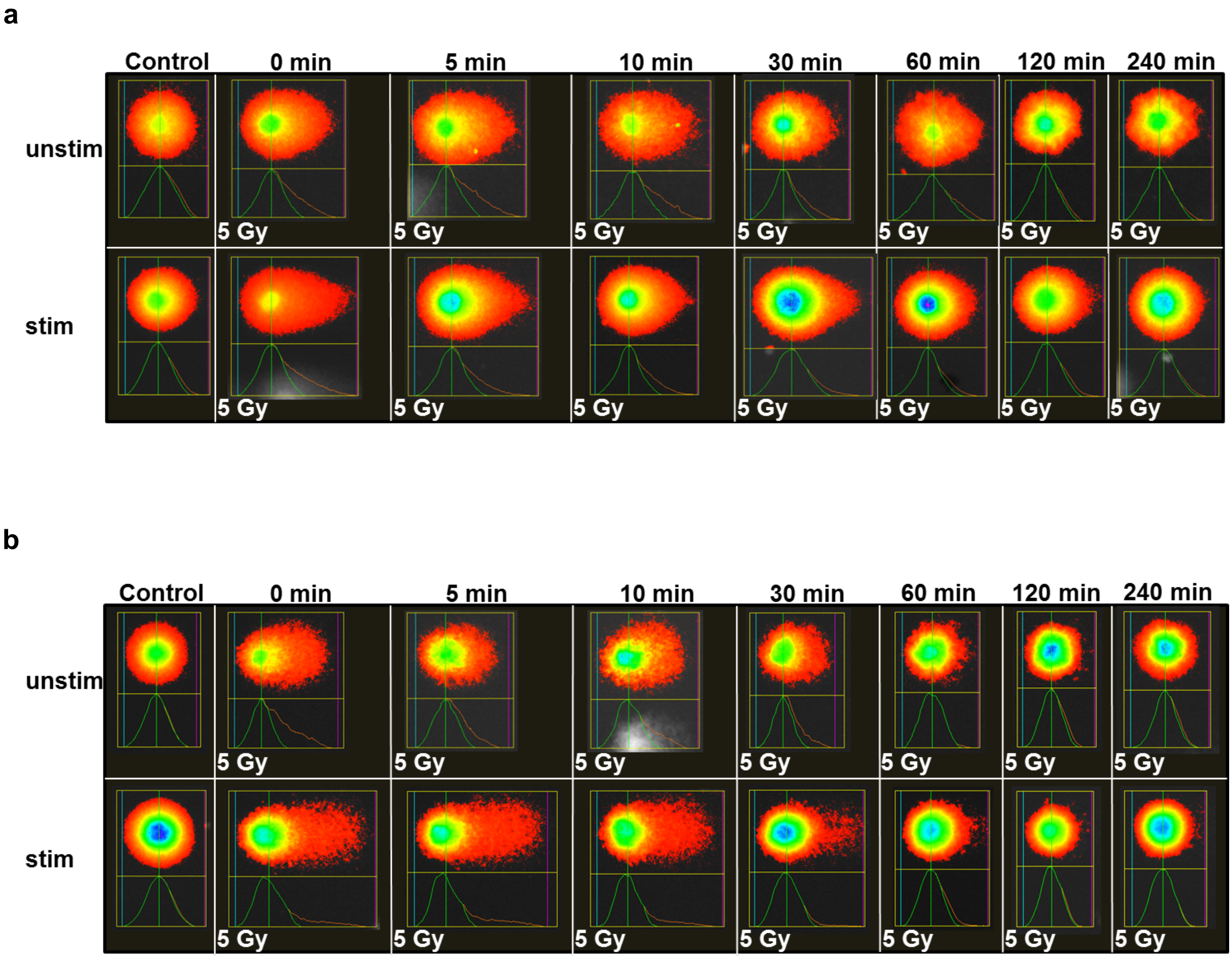

Figure S6 Representative pictures of unstimulated and stimulated PBLC analyzed by the neutral (**a**) and alkaline (**b**) comet assay.

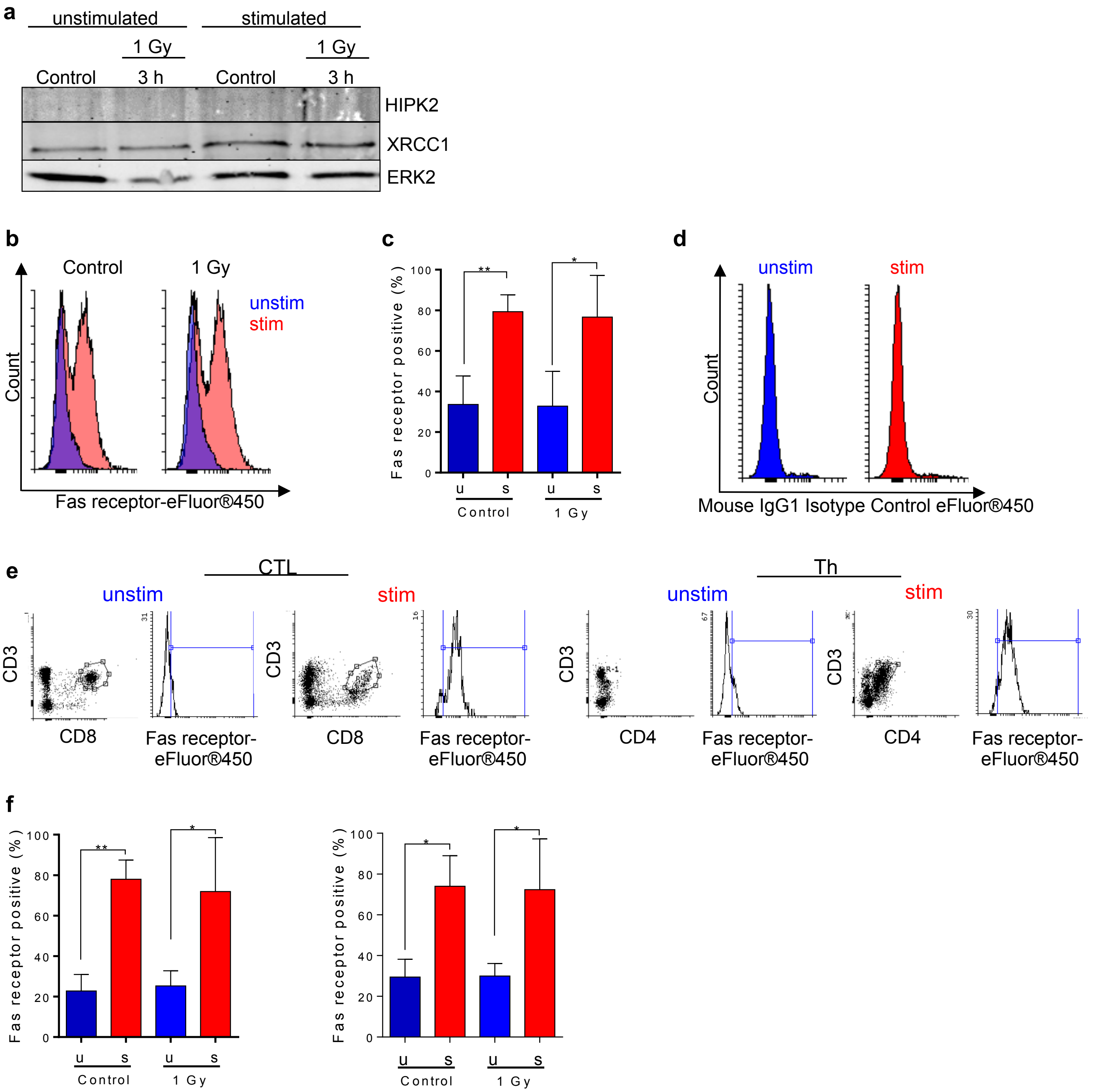

Figure S7 (**a**) Western Blot analysis of HIPK2 and XRCC1 in unstimulated and stimulated PBLC. HIPK2 could not be detected. The base excision repair protein XRCC1 is clearly expressed in lymphocytes. ERK2 was used as loading control. (**b**) Representative histograms (flow cytometry) of fas receptor expression in unstimulated and stimulated PBLC 24 h after -/+ 1 Gy. Fas receptor was analyzed by an eFluor®450 coupled antibody. (**c**) Quantification of fas receptor signal in non-irradiated and irradiated PBLC (n = 3, mean value, SD, t-Test *p<0.05, **p<0.01). Stimulated PBLC showed a significant higher signal of fas receptor expression compared to unstimulated cells, independent from exposure to IR. (**d**) Isotype control showed no eFluor®450 signals (flow cytometry, histograms). (**e**) Representative Dot-Blots and histograms of Fas receptor signals in CD3 CD8 gated CTL (left) and CD3 CD4 gated Th (right) within unstimulated or stimulated PBLC. (**f**) Quantification of fas receptor staining in CTL (left) and Th (right) (n = 3, mean value, SD, t-Test *p<0.05, **p<0.01).

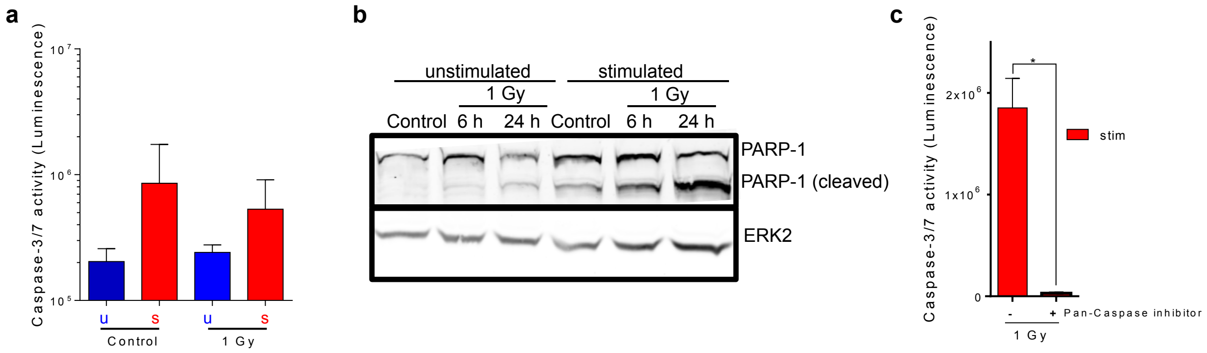

Figure S8 (**a**) Caspase-3/7 activity analyzed 6 h after 1 Gy irradiation in unstimulated and stimulated PBLC (n = 3, mean value, SD). (**b**) Western Blot showing PARP-1 cleavage in stimulated PBLC. (**c**) The caspase-3/7 activity can be significantly reduced by treating the cells with the pan-caspase inhibitor (as shown by the caspase-3/7 activity assay).

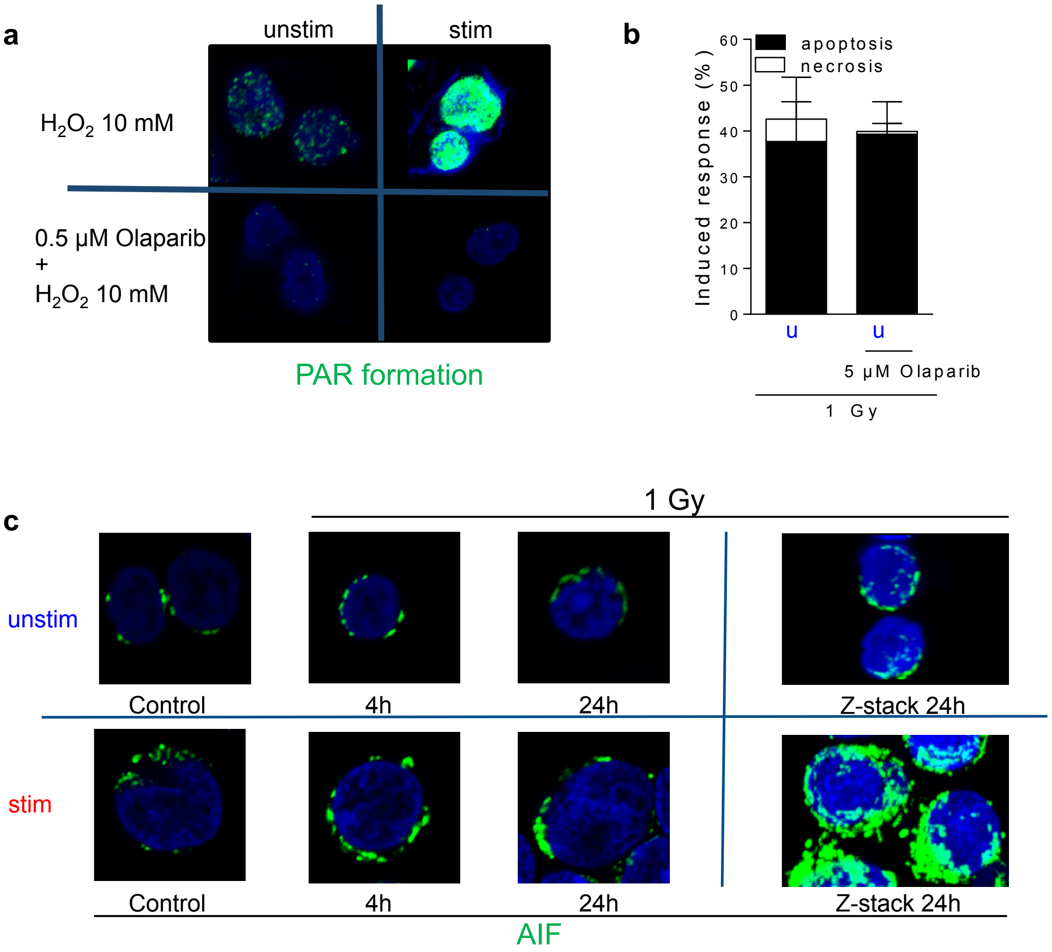

Figure S9 (**a**) Immunostaining of PAR (green) in PBLC (nucleus in blue) treated with H_2O_2 or treated with Olaparib prior H_2O_2 to inhibit PARP-1. PAR formation is visible after H_2O_2 treatment in unstimulated and stimulated PBLC. Olaparib clearly reduced H_2O_2 induced PAR formation, which indicates it's efficiency for PARP-1 inhibition in lymphocytes. (**b**) A high concentration of olaparib (5 µM) had no influence on irradiation induced cell death in unstimulated PBLC. (**c**) Immunostaining of AIF (apoptosis inducing factor) in PBLC. PAR formation results in AIF translocation from mitochondria to the nucleus and is a specific hint for a cell death called Parthanatos. Neither in unstimulated nor in stimulated PBLC a translocation of AIF into the nucleus could be determined after 4 and 24 h post 1 Gy irradiation. 3-dimensional pictures (so called Z-stacks, right side) point out stronger AIF signals around the nucleus in stimulated than unstimulated PBLC (a and c pictures recorded by LSM).

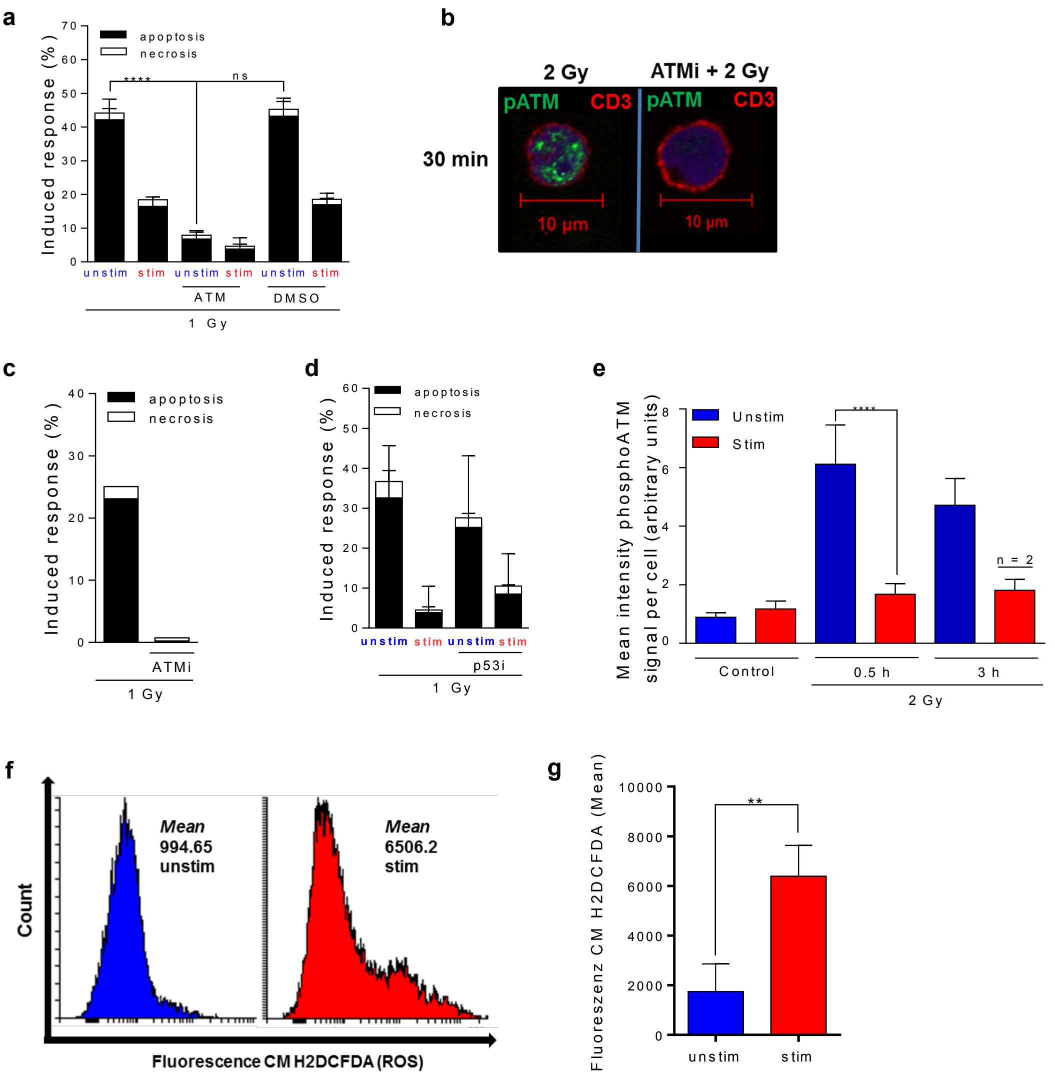

Figure S10 (**a**) A lower concentration of the ATM inhibitor KU60019 (3 µM) also protects unstimulated PBLC from IR induced cell death (Annexin V/PI staining 24 h after IR). DMSO showed no effect on irradiation induced cell death (n = 3, mean value, one-way Anova (Dunnett), ****p<0.0001, ns = not significant). (**b**) Testing the efficiency of the ATM inhibitor KU60019 (3 µM) by immunostaining of phosphoATM (LSM). CD3 (red) T cells (within unstimulated PBLC) showed 30 min after 2 Gy no phosphoATM (green) signals when treated additionally with the ATM inhibitor. (**c**) The alternative ATM inhibitor KU55933 (10 µM) also reduced cell death in unstimulated PBLC (n = 1). (**d**) Increasing the concentration of p53 inhibitor pifithrin-α (100 µM) showed no effect on IR induced cell death of PBLC (n = 2). (**e**) Quantification of phosphATM intensity (immunostaining, LSM) in unstimulated and stimulated CD3 T cells 0.5 and 3 h after 2 Gy (n = 2 – 3, mean value, SD, t-Test, ****p>0.0001). (**f**) Analyzing intracellular ROS in unstimualted and stimulated PBLC with the dye CM H2DCFDA by flow cytometry (representative histograms). (**g**) Quantification of data from (**f**) indicates significantly more intracellular ROS in CD3/CD28 stimulated PBLC compared to unstimulated cells (n = 3. mean value, SD, t-Test, **p<0.01).

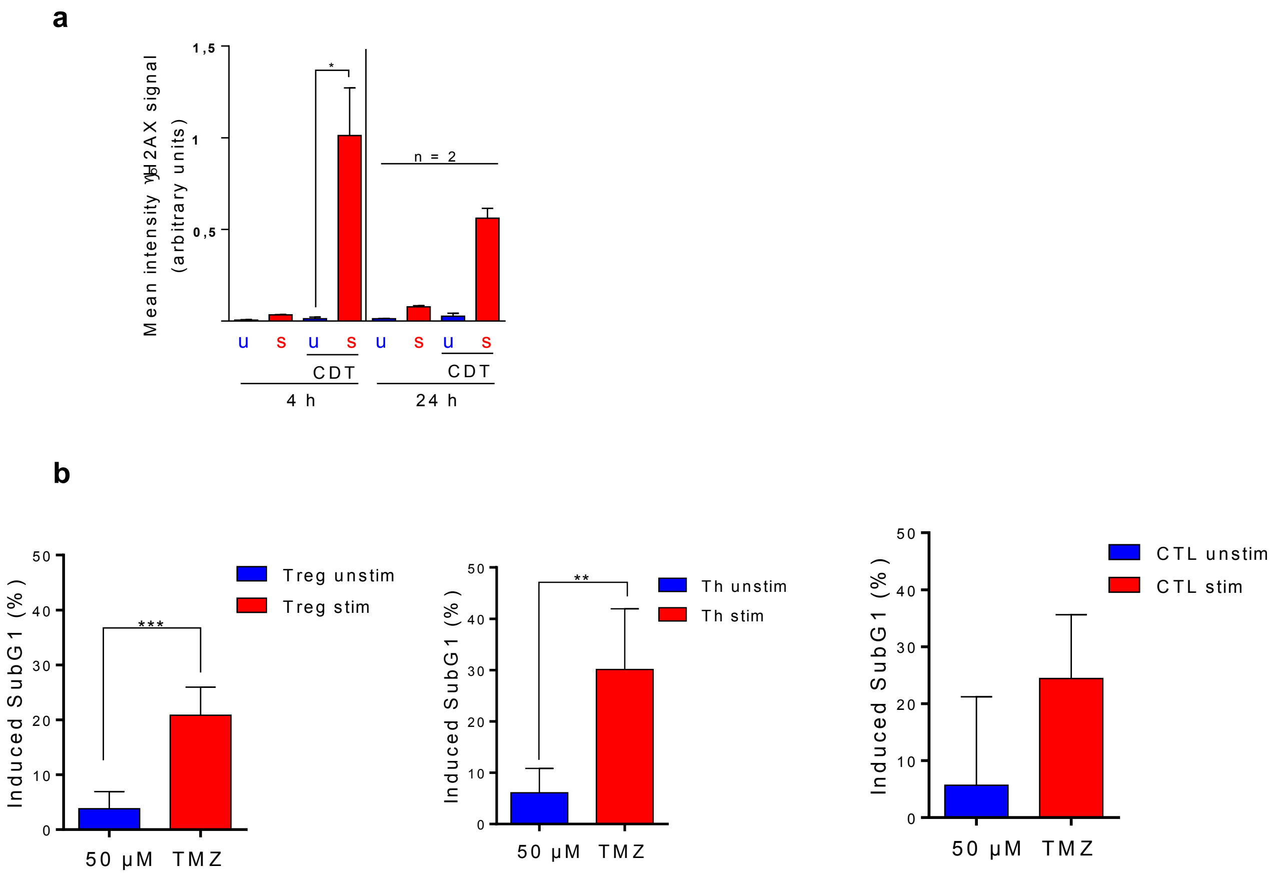

Figure S11 (**a**) Quantification of γH2AX signal in CD3 T cells (mean intensity, immunostaining, LSM) in unstimulated versus stimulated PBLC after CDT treatment (n = 2 - 3, mean value, SEM, t-Test, *p<0.05). (**b**) SubG1 staining of magnetic bead isolated unstimulated or stimulated Treg, Th and CTL 72 h after treatment with 50 µM TMZ and depletion of MGMT with O^6benzylguanine, flow cytometry, n = 5, mean value, t-Test, **p<0.01, ***p<0.001).
